# Adolescent stress and alcohol are associated with CX_3_CR1-linked endocrine–cardiac signatures and anxiety-like behavior in mice

**DOI:** 10.3389/fphar.2026.1850815

**Published:** 2026-06-24

**Authors:** Dina Medina-Vera, Laura Martín-Chaves, Laura Sánchez-Marín, María Díaz-Ottaviano, Ana L. Gavito, Bárbara Pozo-Vilumbrales, Lucía Beltrán-Camacho, Olga Popova, Miguel Romero-Cuevas, Borja Fernández, Jorge Rodríguez-Capitán, Fernando Rodríguez de Fonseca, Manuel F. Jiménez-Navarro, Antonia Serrano, Francisco Javier Pavón-Morón

**Affiliations:** 1 IBIMA Plataforma Bionand (Instituto de Investigación Biomédica de Málaga), Málaga, Spain; 2 Facultad de Medicina, Universidad de Málaga, Málaga, Spain; 3 Unidad Clínica de Cardiología y Cirugía Cardiovascular, Hospital Universitario Virgen de La Victoria, Málaga, Spain; 4 Centro de Investigación Biomédica en Red de Enfermedades Cardiovasculares (CIBERCV), Instituto de Salud Carlos III, Madrid, Spain; 5 Department of Neurobiology, Care Sciences and Society (NVS), Karolinska Institutet, Solna, Sweden; 6 Unidad de Gestión Clínica de Salud Mental, Hospital Regional Universitario de Málaga, Málaga, Spain; 7 Departamento de Biología Animal, Facultad de Ciencias, Universidad de Málaga, Málaga, Spain; 8 Unidad de Gestión Clínica de Neurología, Hospital Regional Universitario de Málaga, Málaga, Spain

**Keywords:** adolescence, alcohol, fractalkine, heart, mouse, stress, troponin

## Abstract

**Background:**

Adolescence is a vulnerable window in which stress and alcohol exposure can induce long-lasting neuroimmune, endocrine, and cardiac molecular alterations. The fractalkine axis (CX_3_CL1/CX_3_CR1) regulates neuron–microglia signaling and inflammatory responses, but its role in linking adolescent stress/alcohol exposure with adult anxiety-like behavior and cardiac biomarker/molecular signatures remains unclear.

**Aims:**

To test whether genetic or pharmacological disruption of CX_3_CR1 modifies adult anxiety-like behavior, circulating biomarkers, and cardiac transcriptional responses after adolescent stress and/or alcohol exposure.

**Methods:**

Male and female C57BL/6J wild-type (WT) and CX_3_CR1 knockout (KO) mice were exposed to restraint stress (90 min) and/or alcohol (2 g/kg, 14 days) during adolescence. In adulthood, elevated plus maze (EPM) behavior was assessed; plasma cTnI/cTnT, CX_3_CL1, ACTH, and corticosterone were quantified; and cardiac mRNA expression of chemokines, inflammatory receptor/NF-κB-related genes, glucocorticoid/mineralocorticoid receptor-related genes, and RAAS-related transcripts was profiled. WT mice received the CX_3_CR1 antagonist AZD8797 (20 mg/kg, i.p.) for behavioral validation.

**Results:**

CX_3_CR1 loss or blockade was associated with increased anxiety index and reduced open-arm exploration under selected exposure conditions. CX_3_CR1 KO mice showed higher plasma cTnI/cTnT, CX_3_CL1, and ACTH, and lower corticosterone, stress and alcohol were also associated with lower corticosterone. In cardiac tissue, alcohol exposure, mainly under the no-stress condition, increased *Cx3cl1*, *Ccl2*, *Ackr3*, and selected RAAS-related transcripts, whereas CX_3_CR1 deficiency was associated with broadly lower expression across chemokine, NF-κB-related, mineralocorticoid receptor-related, and RAAS-related targets. Correlation analyses indicated a chemokine/*Cxcl12*-anchored inflammatory–RAAS transcriptional architecture in WT mice that was attenuated and shifted toward glucocorticoid/mineralocorticoid receptor–NF-κB/IκBα–RAAS-related associations in CX_3_CR1 KO mice.

**Conclusion:**

These findings suggest that CX_3_CR1 signaling contributes to the long-term coordination of behavioral stress-related responses, HPA-axis activity, circulating cardiac injury biomarkers, and cardiac inflammatory/RAAS-related transcriptional programs after adolescent stress and alcohol exposure. Because cardiac function and histopathology were not assessed, these results should be interpreted as evidence of cardiac biomarker and molecular vulnerability rather than definitive myocardial pathology.

## 1 Introduction

CX_3_CL1, also known as fractalkine, is a chemokine that exists as both a membrane-bound adhesion molecule and a soluble chemoattractant. Its canonical receptor, CX_3_CR1, is encoded by the *Cx3cr1* gene and functions as a chemokine receptor involved in CX_3_CL1-dependent immune-cell adhesion, chemotaxis, and inflammatory signaling (Bazan et al., 1997; Imai et al., 1997). In the central nervous system (CNS), CX_3_CR1 is highly expressed by microglia, whereas CX_3_CL1 is predominantly produced by neurons, forming a major neuron–microglia signaling pathway. Through this pathway, the CX_3_CL1/CX_3_CR1 axis is involved in microglial activation, synaptic remodeling, and neuroimmune surveillance (Jung et al., 2000; Cardona et al., 2006; Paolicelli et al., 2011; 2014; Sheridan and Murphy, 2013). Outside the CNS, CX_3_CR1 is expressed by monocytes/macrophages, subsets of cytotoxic T cells, natural killer cells, and cardiovascular immune-cell populations, where it contributes to inflammatory recruitment, endothelial–immune interactions, myocardial ischemia–reperfusion injury, and post-injury remodeling (Combadière et al., 2003; Boag et al., 2015; Spray et al., 2021; Loh et al., 2023). This dual neuroimmune and cardiovascular localization makes CX_3_CR1 a biologically plausible candidate pathway linking stress-related neuroimmune alterations with endocrine and cardiac molecular vulnerability. Given this neuroimmune and cardiovascular distribution, alterations in the CX_3_CL1/CX_3_CR1 axis have been associated with abnormal stress responses, mood and anxiety disorders, and immune dysregulation (Hellwig et al., 2016; Milior et al., 2016; Winkler et al., 2017).

Adolescence constitutes a particularly vulnerable developmental window during which exposure to stress and alcohol can induce long-term behavioral and physiological consequences (Eiland and Romeo, 2013; Tottenham and Galván, 2016; Sánchez-Marín et al., 2022b; 2022a). Acute stressors activate the hypothalamic–pituitary–adrenal (HPA) axis and promote microglial reactivity, while alcohol exposure during adolescence disrupts neuroendocrine and immune homeostasis (Boden and Fergusson, 2011; Walter et al., 2017). These neurodevelopmental perturbations may persist into adulthood, increasing vulnerability to anxiety-like behavior and impaired stress adaptation (Sánchez-Marín et al., 2022b; 2022a); however, few studies have directly tested whether early-life exposure to stress and alcohol jointly shapes long-term neuroendocrine and cardiac molecular vulnerability, and current support largely derives from separate lines of evidence for adolescent stress and alcohol exposure (Piano, 2017; Arora et al., 2022).

Alcohol exposure is also closely linked to neuroimmune activation, particularly during development and adolescence, including microglial activation and altered inflammatory signaling, which may contribute to persistent alcohol-related behavioral and physiological vulnerability (Crews et al., 2015; Doremus-Fitzwater and Deak, 2022). Although direct evidence linking CX_3_CL1/CX_3_CR1 to alcohol responses remains more limited than for stress, recent studies in alcohol-preferring rats and prenatal–lactational alcohol exposure models support the involvement of fractalkine signaling in alcohol-related neuroimmune, neuroendocrine, and cardiovascular alterations (Balan et al., 2024; Medina-Vera et al., 2026). Our group previously showed that CX_3_CR1 deficiency alters the neuroimmune response to adolescent stress and alcohol. Genetic deletion or pharmacological blockade of CX_3_CR1 was associated with altered stress-coping strategies, disrupted hypothalamic expression of corticotropin-releasing hormone (CRH) and neuropeptide Y (NPY) pathways, altered HPA-axis regulation, and exaggerated systemic inflammation (Medina-Vera et al., 2025). These findings highlighted a protective role for CX_3_CL1/CX_3_CR1 signaling in maintaining stress-related neuroimmune homeostasis and controlling inflammation during adolescence.

Beyond the CNS, the CX_3_CL1/CX_3_CR1 axis has been increasingly implicated in cardiovascular pathology. In patients with myocardial infarction, higher circulating CX_3_CL1 has been associated with poorer myocardial recovery and major adverse cardiac events (Xu et al., 2019); mechanistically, CX_3_CR1-dependent lymphocyte dynamics are linked with microvascular obstruction and subsequent adverse left-ventricular remodeling (Boag et al., 2015; Spray et al., 2021). In preclinical models, neutralizing fractalkine or inhibiting CX_3_CR1 attenuates infarct inflammation, reduces infarct size, and limits adverse remodeling (Gu et al., 2015; Loh et al., 2023), while genetic deficiency of CX_3_CR1 or CX_3_CL1 reduces atherosclerotic lesion formation (Combadière et al., 2003; Teupser et al., 2004). Clinically, circulating CX_3_CL1 is elevated in coronary artery disease and predicts mortality in advanced heart failure (Damås et al., 2005; Richter et al., 2012), and higher CX_3_CL1 levels are associated with incident myocardial infarction and all-cause mortality in chronic kidney disease (Shah et al., 2015). In abstinent individuals with alcohol and cocaine use disorders, we recently observed elevated cardiac troponins that were associated with plasma CX_3_CL1, supporting a link between fractalkine dysregulation and circulating cardiac injury biomarkers (Porras-Perales et al., 2025).

These findings indicate that the CX_3_CL1/CX_3_CR1 axis may serve as a mechanistic link between stress-related neuroimmune alterations, substance exposure, and cardiovascular vulnerability. We hypothesized that perturbation of fractalkine signaling modulates the impact of adolescent stress and/or alcohol on adult anxiety-like behavior, HPA-axis activity, circulating cardiac injury biomarkers, and cardiac inflammatory/RAAS-related transcriptional programs. Accordingly, we aimed to (i) evaluate anxiety-related behavior after adolescent stress and/or alcohol in wild-type (WT) and CX_3_CR1 knockout (KO) mice; (ii) quantify plasma biomarkers relevant to neuroimmune and cardiovascular pathways; (iii) profile cardiac transcripts across chemokine networks, proinflammatory receptors and NF-κB signaling, stress-hormone receptors, and components of the renin–angiotensin–aldosterone system (RAAS)-related pathways; and (iv) complement genetic findings with pharmacological antagonism of CX_3_CR1.

The selected physiological and molecular measures were intended to capture complementary levels of long-term stress-related adaptation. Elevated plus maze (EPM)-derived open-arm exploration, anxiety index, and total distance traveled were used as behavioral readouts of anxiety-like behavior and locomotor activity. ACTH and corticosterone were included as endocrine markers of HPA-axis activity and pituitary–adrenal coupling. Circulating cTnI and cTnT were measured as sensitive biomarkers of cardiomyocyte injury, whereas plasma CX_3_CL1 was used as a systemic marker of fractalkine signaling. At the cardiac level, gene-expression analyses were organized into functional modules related to chemokine signaling, proinflammatory receptor/NF-κB pathways, glucocorticoid/mineralocorticoid receptor-related signaling, RAAS-related regulation, and cardiac stress/remodeling.

We hypothesized that CX_3_CR1 signaling contributes to long-term adaptation to adolescent stress and alcohol exposure by coordinating behavioral stress-related responses, HPA-axis activity, circulating endocrine/cardiac biomarkers, and cardiac inflammatory/RAAS-related transcriptional programs. In this framework, anxiety-like behavior was considered an integrated behavioral readout of altered stress adaptation, whereas ACTH/corticosterone, circulating troponins/CX_3_CL1, and cardiac mRNA profiles were used to assess peripheral neuroendocrine and cardiac molecular signatures. Specifically, we expected CX_3_CR1 deficiency to be associated with increased anxiety-like behavior, altered ACTH/corticosterone balance, elevated circulating cardiac injury biomarkers, and disrupted cardiac inflammatory/RAAS-related gene-expression patterns, while pharmacological CX_3_CR1 antagonism in WT mice was expected to partially reproduce the behavioral phenotype observed in CX_3_CR1-deficient mice.

## 2 Methodology

### 2.1 Ethics statement and animals

All procedures were conducted in accordance with ARRIVE guidelines (Kilkenny et al., 2010) and the principles of replacement, reduction, and refinement (the “3Rs”), complying with the European Communities Council Directive 2010/63/EU and Council Directive 86/609/EEC of 24 November 1986, as well as Spanish National and Regional Guidelines for Animal Experimentation (Real Decreto 53/2013). The experimental protocols were approved by the Local Ethical Committee for Animal Research at the University of Malaga (CEUMA #59-2020-A). Every effort was made to minimize animal suffering and reduce the number of animals used.

C57BL/6J mice were used as WT controls and maintained by crossing WT mice (The Jackson Laboratory, Bar Harbor, ME, United States). Additionally, we used the Cx3cr1^GFP^ knock-in/knock-out mouse line (C57BL/6J genetic background), which expresses an enhanced green fluorescent protein (E*GFP*) sequence replacing the first 390 bp of the coding exon (exon 2) of the chemokine (C-X3-C motif) receptor 1 *Cx3cr1* gene (Jung et al., 2000). The Cx3cr1^GFP^ knock-in/knock-out mice were maintained by crossing homozygous transgenic mice (The Jackson Laboratory, Bar Harbor, ME, United States). Both WT and CX_3_CR1 KO mice were housed in groups of three to four per cage on a 12-h light/dark cycle (lights on at 07:00 h) with water and food provided *ad libitum* and appropriate environmental enrichment.

### 2.2 Experimental groups and procedures

#### 2.2.1 WT and CX_3_CR1 KO genetic cohort

**FIGURE 1:**
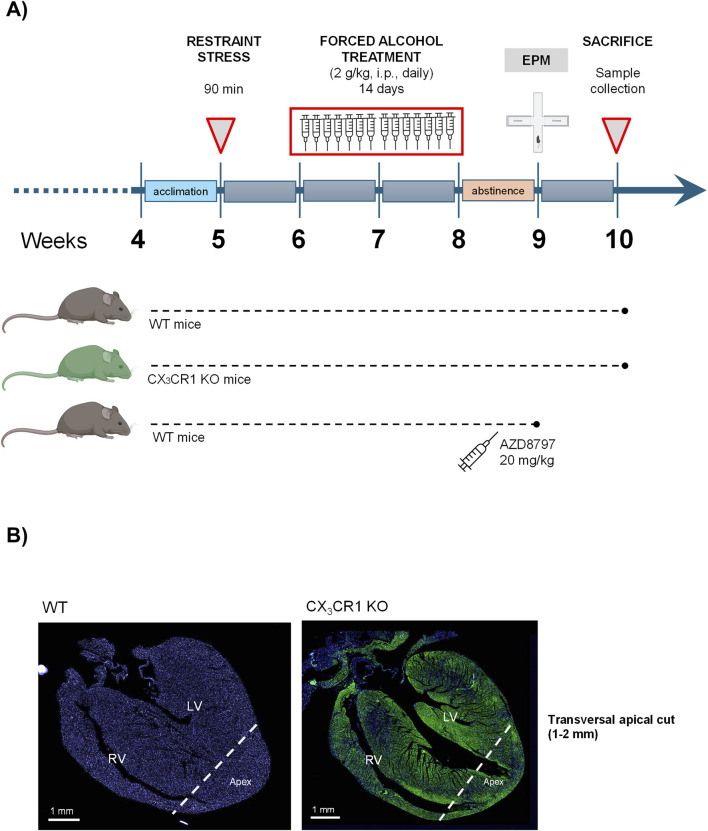
Experimental design and representative long-axis heart sections used for apical gene-expression sampling. **(A)** Timeline and experimental schedule. At 4 weeks of age, mice were acclimatized to the testing/treatment environment for 1 week. WT and CX_3_CR1 KO mice were then assigned to stress or non-stress conditions; stressed mice underwent acute restraint stress (90 min). One week later, mice received daily i.p. saline or ethanol (2 g/kg; 10 mL/kg) for 14 days. Anxiety-like behavior and exploratory activity were assessed in the elevated plus maze (EPM) 7 days after the final injection. Mice were euthanized 2 weeks after the final injection, and blood and hearts were collected. In an additional WT cohort used for pharmacological validation, AZD8797 (20 mg/kg, i.p.) was administered 30 min before EPM testing (no samples were collected from this cohort). **(B)** Representative long-axis heart sections from WT and CX_3_CR1-deficient *Cx3cr1* ^GFP^ knock-in/knock-out (CX_3_CR1 KO) mice. Nuclei are shown in blue and GFP fluorescence (green) reports *Cx3cr1*-expressing cells in CX_3_CR1 KO hearts. The dashed line indicates the transverse plane used to obtain a 1–2 mm apical ring (apex) for cardiac gene-expression analyses. LV, left ventricle; RV, right ventricle.

Male and female WT and CX_3_CR1 KO mice were randomly assigned to one of four experimental groups (n = 6–10 per group; sex-balanced, three to five males and three to five females): control (non-stressed, saline-treated), stress (stressed, saline-treated), alcohol (non-stressed, alcohol-treated), and stress + alcohol (stressed, alcohol-treated mice) groups. Stress and alcohol exposures followed procedures described previously (Medina-Vera et al., 2025). The experimental timeline and procedural details are summarized in Figure 1A.

#### 2.2.2 Pharmacological CX_3_CR1 antagonism cohort

To complement the genetic model, CX_3_CR1 was pharmacologically inhibited in WT mice using AZD8797 (CAS 911715-90-7; Axon Medchem, Groningen, Netherlands), a potent, selective, allosteric, non-competitive CX_3_CR1 antagonist. AZD8797 has a dissociation rate constant (Koff) of 0.042 min^-1^, corresponding to an approximate receptor dissociation half-life of 17 min (Cederblad et al., 2016).

First, young adult WT mice aged 9–11 weeks that had not been exposed to stress or alcohol were randomly assigned to receive vehicle or AZD8797 at 0.2, 2, or 20 mg/kg to identify the dose that most closely phenocopied the EPM behavioral profile of untreated CX_3_CR1 KO mice (Supplementary Figure S1). Groups comprised n = 6 mice per dose and were sex-balanced, with three males and three females per group. AZD8797 was dissolved in sterile saline and administered i.p. at 10 mL/kg, 30 min before the EPM testing.

After identifying 20 mg/kg as the selected dose, WT mice from an independent cohort were randomly assigned to control, stress, alcohol, or stress + alcohol groups (n = six to seven per group; sex-balanced, three to four males and three females). One week after the final alcohol or saline administration, mice aged 9 weeks received AZD8797 (20 mg/kg, i.p.) or vehicle, and were tested in the EPM 30 min later. Vehicle-treated mice were included as contemporaneous pharmacological controls. This pharmacological validation cohort was independent from the WT and CX_3_CR1 KO genetic cohort and was designed to assess whether acute CX_3_CR1 antagonism reproduced aspects of the behavioral phenotype observed in CX_3_CR1-deficient mice; therefore, no blood or cardiac tissue samples were collected, and AZD8797-related analyses were restricted to EPM outcomes.

#### 2.2.3 Acute restraint stress

Five-week-old mice assigned to the stress and stress + alcohol groups were placed in appropriately sized Plexiglass restraint tubes for a continuous 90-min session during the light phase, between 08.00 h and 09:30 h, and then returned to their home cages (Sánchez-Marín et al., 2022b). This procedure was not repeated on subsequent days. Non-stressed mice in the control and alcohol groups remained undisturbed in their home cages throughout the procedure.

#### 2.2.4 Forced alcohol treatment and body-weight monitoring

Mice in the alcohol and stress + alcohol groups received daily intraperitoneal (i.p.) ethanol injections (2 g/kg; 10 mL/kg) for 14 consecutive days, beginning at 6 weeks of age, between 08.00 h and 09:30 h (Anderson et al., 2016). Control and stress mice received i.p. saline on the same schedule as the alcohol injections to match handling and injection exposure across groups. Body weight was recorded immediately before the ethanol/saline administration at baseline (day 0) and on treatment days 3, 6, 9, and 12, and again on day 15, 1 day after the final injection. Following the final administration, mice were left undisturbed in their home cages until behavioral testing.

### 2.3 Elevated plus maze

One week after the final alcohol or saline administration, 9-week-old WT and CX_3_CR1 KO mice were weighed and tested in the EPM to assess anxiety-like behavior and locomotor activity. In the pharmacological validation cohort, vehicle or AZD8797 was administered 30 min before EPM testing, as described above.

The apparatus consisted of a black, cross-shaped maze with four arms (16 × 5 cm) extending from a central square (5 × 5 cm). Two opposing arms were enclosed by vertical walls (closed arms), whereas the other two arms were unprotected (open arms). The maze was elevated 30 cm above the floor, and testing was conducted under dim lighting (30 lx). At the beginning of each 5-min session, mice were placed in the central square facing an open arm.

EPM testing was performed during the light phase, between 08:00 h and 10:00 h. Behavior was recorded and tracked using automated software (SMART; Panlab, Barcelona, Spain). Time spent in the open and closed arms (expressed as a percentage of total test duration) and the number of arm entries were quantified. An arm entry was defined as the placement of all four paws into the corresponding arm zone. In addition, total distance traveled (m) was extracted from the tracking output as a complementary index of locomotor activity. An anxiety index ranging from 0 (low anxiety) to 1 (high anxiety) was also calculated based on the following formula: 1 − [(open-arm time/total time) + (open-arm entries/total arm entries)]/2 (Yeom et al., 2020).

Zones were defined *a priori* and kept constant across sessions, and automated tracking was verified by manual scoring in a subset of videos by two independent observers. Behavioral scoring and data analysis were performed under double-blind conditions.

### 2.4 Sample collection and tissue processing

For the WT and CX_3_CR1 KO genetic cohort, 1 week after the EPM (i.e., 2 weeks after the final alcohol or saline administration), mice were deeply anesthetized with sodium pentobarbital (50 mg/kg, i.p.); depth was confirmed by the absence of the pedal withdrawal reflex. As a terminal procedure, guillotine decapitation was performed. Trunk blood was collected immediately, followed by a rapid thoracotomy to excise the heart.

Blood was collected into BD Vacutainer™ plastic blood collection tubes containing K_2_EDTA (Becton, Dickinson and Company, Franklin Lakes, NJ, United States) and centrifuged at 2,000×g for 15 min at 4 °C. Plasma was aliquoted and stored at −80 °C.

Hearts were excised within ∼one to two min, immersed in ice-cold phosphate-buffered saline (PBS), and gently rinsed to remove blood. On a dissection surface (4 °C), pericardium, great vessels, and epicardial fat were removed. With the apex oriented distally, a transverse cut just proximal to the apex tip yielded an apical ring 1–2 mm thick, which was stored at −80 °C (Figure 1B).

### 2.5 Plasma biomarker measurements

Plasma levels of cardiac troponins I and T (cTnI and cTnT), fractalkine (CX_3_CL1), adrenocorticotropic hormone (ACTH), and corticosterone (CORT) were quantified in both genotypes using multiplex bead-based assays and single-analyte ELISAs. These biomarkers were selected to assess complementary circulating signatures: cTnI and cTnT as indicators of cardiomyocyte injury, CX_3_CL1 as a systemic readout of fractalkine signaling, and ACTH/corticosterone as endocrine markers of HPA-axis activity. Specifically, cTnT and cTnI were quantified using the MILLIPLEX® Mouse Cardiovascular Disease (CVD) Magnetic Bead Panel 2 - Cardiovascular Disease Multiplex Assay (Cat. #MCVD2MAG-77K, Millipore, Merck, Darmstadt, Germany); CX_3_CL1 was quantified using the Mouse Fractalkine/CX3CL1 ELISA Kit (Cat. #EMCX3CL1X5, Invitrogen, Thermo Fisher Scientific, Waltham, MA, United States); ACTH was quantified using the MILLIPLEX® Mouse Pituitary Magnetic Bead Panel - Endocrine Multiplex Assay (Cat. #MPTMAG-49K, Millipore, Merck, Darmstadt, Germany); and corticosterone was quantified using a Corticosterone ELISA kit (Cat. #AB108821, Abcam, Cambridge, UK). All determinations were performed according to the manufacturer’s instructions.

Plates for cTnT and cTnI were run on a Bio-Plex MAGPIX™ Multiplex Reader with Bio-Plex Manager™ MP Software (Luminex, Austin, TX, United States), whereas plates for CX_3_CL1, ACTH, and corticosterone were analyzed on an Accuris SmartReader^TM^ 96 microplate absorbance reader (Merck, Darmstadt, Germany). All samples were assayed in duplicate, and concentrations were expressed as pg/mL after correction for the corresponding dilution factors. Intra-assay coefficient of variation (CV) ranges, calculated from duplicate measurements within each plate, were as follows: cTnI, 4%–12%; cTnT, 8%–16%; CX_3_CL1, 6%–15%; ACTH, 8%–18%; and corticosterone, 8%–16%. Because each analyte was measured in a single 96-well plate/assay run, experimental inter-assay CVs were not applicable (manufacturer-reported assay performance was reviewed but was not included as experimental inter-assay variability). Concentrations for samples with absorbance values below the limit of detection but above background were set to half of the minimum concentration interpolated from the corresponding standard curve (Whitcomb and Schisterman, 2008; Jiménez-López et al., 2024).

### 2.6 Cardiac gene expression

Total RNA was isolated from apical ring sections (∼25 mg) using TRIzol™ (Invitrogen/Thermo Fisher Scientific, Waltham, MA, United States) following the manufacturer’s instructions. RNA was further cleaned using an RNeasy MinElute Cleanup Kit with on-column DNase I digestion (Qiagen) and quantified by spectrophotometry (A260/280 between 1.8 and 2.0). Reverse transcription was performed using 1 μg RNA (Transcriptor RT, Roche Applied Science, Mannheim, Germany).

#### 2.6.1 TaqMan-based cardiac gene-expression panel

Cardiac transcripts were selected *a priori* to represent predefined biological pathways relevant to the study hypothesis and to provide molecular information on inflammatory, neuroendocrine, and RAAS-related cardiac responses: fractalkine axis (*Cx3cl1*, *Cx3cr1*); chemokines and receptors (*Ccl2*, *Ccl5*, *Ccl11*, *Cxcl12*, *Ccr2*, *Cxcr4*, *Ackr3*); proinflammatory cytokine receptors (*Tnfrsf1a*, *Tnfrsf1b*, *Il1r1*); NF-κB signaling (*Nfkb1*, *Nfkbia*); stress-hormone receptors (*Nr3c1*, *Nr3c2*); and RAAS-related genes (*Agtr1a*, *Ace2*, *Mas1*). *Actb* served as the reference gene (Supplementary Table S1). Real-time qPCR reactions were run on a CFX96^TM^ Real-Time PCR Detection System (Bio-Rad, Hercules, CA, United States) using FAM-labeled TaqMan® Gene Expression Assays (Thermo Fisher Scientific, Waltham, MA, United States). Expression was normalized to *Actb* levels.

#### 2.6.2 Targeted qPCR analysis of cardiac stress and remodeling markers

To complement the troponin and cardiac transcriptional data, we performed an additional targeted analysis of cardiac stress and remodeling markers in the same RNA samples. Specifically, we quantified *Nppa*, encoding atrial natriuretic peptide (ANP); *Nppb*, encoding B-type natriuretic peptide (BNP); and *Myh7*, encoding β-myosin heavy chain (β-MHC). These markers were selected to provide additional molecular information on cardiac stress/remodeling-related transcriptional responses, rather than direct evidence of structural remodeling. *Nppa* and *Nppb* were included as natriuretic peptide-related markers that provide molecular context for circulating troponin findings, whereas *Myh7* was included as an additional remodeling-related marker.

This analysis was performed using primer-based qPCR, whereas the main cardiac gene-expression panel used FAM-labeled TaqMan® assays; therefore, these data were analyzed and reported separately. qPCR reactions were performed using primer-based amplification chemistry on a CFX96™ Real-Time PCR Detection System (Bio-Rad, Hercules, CA, United States). *Gapdh* was used as the reference gene. Relative expression was calculated using the 2^−ΔCt^ method. Primer sequences are provided in Supplementary Table S2.

### 2.7 Statistical analysis

Data are presented as mean ± SEM unless otherwise stated. Body weight gain from the onset of alcohol or saline exposure to EPM testing was analyzed using one-way analysis of variance (ANOVA) within each genotype. Body weight trajectory across the alcohol or saline exposure period was analyzed using repeated-measures ANOVA.

For the WT and CX_3_CR1 KO genetic cohort, behavioral, plasma biomarker, and cardiac molecular outcomes were analyzed using three-way ANOVA with stress exposure (present vs. absent), alcohol exposure (alcohol vs. saline), and genotype (WT vs. CX_3_CR1 KO) as fixed factors. This factorial model was selected as the primary analytical framework because the experimental design included two binary adolescent exposures and genotype. This approach allowed us to estimate the independent main effects of stress, alcohol, and genotype, as well as stress × alcohol, stress × genotype, alcohol × genotype, and stress × alcohol × genotype interactions.

For the pharmacological validation cohort, EPM outcomes were analyzed using three-way ANOVA with stress exposure (present vs. absent), alcohol exposure (alcohol vs. saline), and pharmacological treatment (AZD8797 vs. vehicle) as fixed factors.

When significant interactions were detected, Tukey’s *post hoc* test was applied for multiple comparisons among experimental groups. The four exposure groups (i.e., control, stress, alcohol, and stress + alcohol) were retained for graphical representation and *post hoc* interpretation. Because several plasma biomarkers exhibited high dispersion and departures from normality, values were log-transformed prior to ANOVA to better meet model assumptions.

Sex-stratified analyses were not performed as primary analyses; instead, males and females were balanced across groups to minimize confounding while preserving power for genotype-, exposure-, and treatment-related effects. Associations between plasma biomarkers and cardiac mRNA were assessed using Spearman’s rank correlations. Correlations were computed separately within WT and CX_3_CR1 KO mice, pooling all exposure conditions within each genotype, and are considered exploratory.

Test statistics, degrees of freedom, and *p*-values are reported where applicable. Statistical significance was set at *p* < 0.05. Analyses were performed using GraphPad Prism v9 (GraphPad Software, Inc., La Jolla, CA, United States).

## 3 Results

Results are reported according to the primary factorial models described in the Statistical Analysis section. For the WT and CX_3_CR1 KO genetic cohort, stress, alcohol, and genotype were modeled as independent factors. For the AZD8797 pharmacological validation cohort, stress, alcohol, and treatment were modeled as independent factors. When significant interactions were detected, *post hoc* comparisons were used to clarify group-level differences shown in the figures.

### 3.1 Body weight, anxiety-like behavior, and locomotor activity

#### 3.1.1 Body weight gain in WT and CX_3_CR1 KO mice

Body weight was evaluated as a potential confounding factor in WT and CX_3_CR1 KO mice. Body weight gain from the onset of ethanol/saline administration to EPM testing did not differ significantly among exposure conditions within either genotype, as shown by one-way ANOVA performed separately in WT and CX_3_CR1 KO mice (Supplementary Figures S2A, S2C). In addition, repeated-measures ANOVA of body weight measured at baseline and on days 3, 6, 9, 12, and 15 revealed no significant main effect of exposure condition in either WT or CX_3_CR1 KO mice (Supplementary Figures S2B, S2D, respectively).

#### 3.1.2 Anxiety-like behavior and locomotor activity in WT and CX_3_CR1 KO mice

One week after the last alcohol or saline administration, exploratory behavior was assessed as an index of anxiety-like behavior in adult WT and CX_3_CR1 KO mice using the EPM.

**FIGURE 2:**
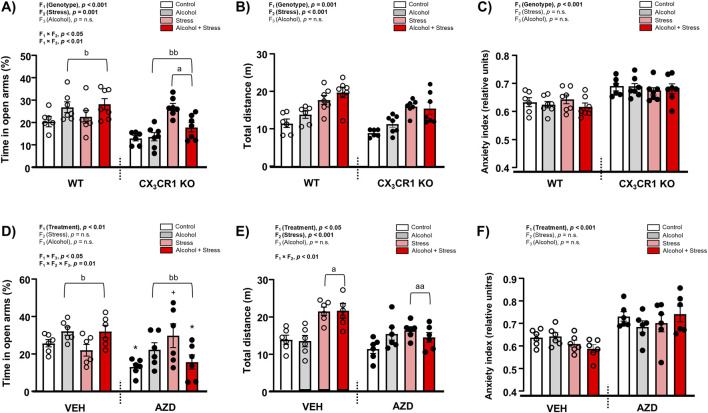
Effects of adolescent stress and/or alcohol exposure on exploratory activity and anxiety-like behavior in adult WT and CX_3_CR1 KO mice in the elevated plus maze. **(A)** Time spent in the open arms (%) in WT and CX_3_CR1 KO mice exposed to stress and/or alcohol. **(B)** Total distance traveled (m) in WT and CX_3_CR1 KO mice exposed to stress and/or alcohol. **(C)** Anxiety index (relative units) in WT and CX_3_CR1 KO mice exposed to stress and/or alcohol. **(D)** Time spent in the open arms (%) in WT mice exposed to stress and/or alcohol after vehicle or AZD8797 (20 mg/kg, **(I)**.p.) administration. **(E)** Total distance traveled (m) WT mice exposed to stress and/or alcohol after vehicle or AZD8797 (20 mg/kg, i.p.) administration. **(F)** Anxiety index (relative units) WT mice exposed to stress and/or alcohol after vehicle or AZD8797 (20 mg/kg, i.p.) administration. Bars represent the mean ± SEM (6–8 mice per group). Data were analyzed using three-way ANOVA with genotype or treatment (F_1_), stress (F_2_), and alcohol (F_3_) as factors. *Post hoc* comparisons are shown only when significant: **(A)** indicates significant differences between stress-exposed and non-stressed conditions within the same genotype or treatment, as applicable; (aa) indicates significant differences between genotypes or treatments under the stress-exposed condition, as applicable; **(B)** indicates significant differences between alcohol-treated and saline-treated conditions within the same genotype or treatment, as applicable; (bb) indicates significant differences between genotypes or treatments under the alcohol-treated condition, as applicable; (*) indicates significant differences vs. the corresponding group of the other genotype or treatment, as applicable; (+) indicates significant differences vs. the control group in the same genotype or treatment, as applicable. n.s = non-significant.

For time spent in the open arms, three-way ANOVA with genotype, stress, and alcohol as factors revealed significant main effects of genotype (F_(1,46)_ = 18.74; *p* < 0.001) and stress (F_(1,46)_ = 12.43; *p* = 0.001), with no significant main effect of alcohol. Significant genotype × stress (F_(1,46)_ = 5.613; *p* = 0.022) and genotype × alcohol (F_(1,46)_ = 11.06; *p* = 0.002) interactions were also detected. These results indicate that CX_3_CR1 deficiency and stress exposure influenced open-arm exploration, and that the effects of stress and alcohol differed according to genotype. *Post hoc* comparisons showed that, in WT mice, alcohol increased open-arm time under the no-stress condition compared with non-stressed/saline-treated WT mice (*p* < 0.05). In CX_3_CR1 KO mice, stress increased open-arm time under saline-treated conditions compared with non-stressed/saline-treated CX_3_CR1 KO mice (*p* < 0.05). Under alcohol exposure, CX_3_CR1 KO mice showed lower open-arm time than WT mice (*p* < 0.05) (Figure 2A).

Total distance traveled was used as an index of locomotor activity. Significant main effects of genotype (F_(1,46)_ = 12.03; *p* = 0.001) and stress (F_(1,46)_ = 54.55; *p* < 0.001) were observed, with no significant main effect of alcohol and no significant interaction terms. Consistent with these main effects, CX_3_CR1 KO mice traveled shorter distances than WT mice, whereas stress-exposed mice traveled greater distances than non-stressed mice (Figure 2B).

To examine the potential influence of locomotor activity on open-arm exploration, we calculated an anxiety index. The factorial model showed a significant main effect of genotype (F_(1,46)_ = 26.11; *p* < 0.001), with CX_3_CR1 KO mice exhibiting a higher anxiety index than WT mice. No significant main effects of stress or alcohol and no significant interaction terms were detected (Figure 2C).

#### 3.1.3 Anxiety-like behavior and locomotor activity in mice treated with CX_3_CR1 antagonist

To complement the genetic findings, CX_3_CR1 was pharmacologically inhibited in WT mice using AZD8797.

For time spent in the open arms, three-way ANOVA using stress, alcohol, and treatment as factors revealed a significant main effect of treatment (F_(1,40)_ = 8.53; *p* = 0.006), together with significant treatment × alcohol (F_(1,40)_ = 4.41; *p* = 0.042) and treatment × stress × alcohol (F_(1,40)_ = 7.24; *p* = 0.010) interactions. These results indicate that AZD8797 treatment influenced open-arm exploration and that the alcohol-related effect differed according to treatment and stress condition. *Post hoc* comparisons were used to examine simple effects across the displayed groups. In vehicle-treated mice, alcohol increased open-arm time under the no-stress condition compared with non-stressed/saline-treated vehicle mice (*p* < 0.05). In AZD8797-treated mice, stress increased open-arm time under saline-treated conditions compared with non-stressed/saline-treated AZD8797 mice (*p* < 0.05). Treatment-related differences were also detected under specific exposure conditions: AZD8797-treated mice showed lower open-arm time than vehicle-treated mice in the non-stressed/saline-treated group (*p* < 0.05) and in the stress + alcohol group (*p* < 0.05) (Figure 2D).

For total distance traveled, significant main effects of treatment (F_(1,40)_ = 6.32; *p* = 0.016) and stress (F_(1,40)_ = 25.04; *p* < 0.001) were observed, together with a significant treatment × stress interaction (F_(1,40)_ = 8.55; *p* = 0.006). These findings indicate that stress exposure increased locomotor activity and that this stress-related effect differed according to AZD8797 treatment. In vehicle-treated mice, stress exposure increased total distance traveled compared with the corresponding non-stressed vehicle-treated mice (*p* < 0.05). Under stress exposure, AZD8797-treated mice traveled shorter distances than vehicle-treated mice under both saline-treated and alcohol-treated conditions (*p* < 0.05) (Figure 2E).

For the anxiety index, the analysis revealed a significant main effect of treatment (F_(1,40)_ = 19.84; *p* < 0.001), with AZD8797-treated mice exhibiting a higher anxiety index than vehicle-treated mice. No significant main effects of stress or alcohol and no significant interaction terms were detected (Figure 2F).

### 3.2 Effects of stress, alcohol, and genotype on plasma biomarkers

**FIGURE 3:**
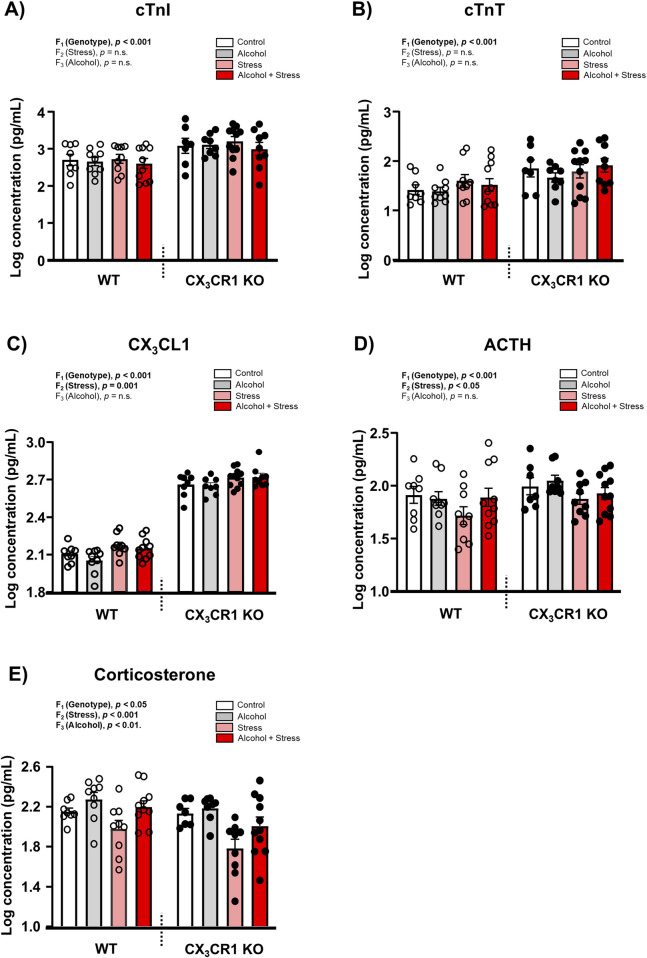
Effects of adolescent stress and/or alcohol exposure on plasma biomarkers in adult WT and CX_3_CR1 KO mice. **(A)** Plasma cTnI levels. **(B)** Plasma cTnT levels. **(C)** Plasma CX_3_CL1 levels. **(D)** Plasma ACTH levels. **(E)** Plasma corticosterone levels. Concentrations (pg/mL) were log10-transformed for analysis and graphical representation. Bars represent the mean ± SEM (7–10 mice per group). Data were analyzed using three-way ANOVA with genotype (F_1_), stress (F_2_), and alcohol (F_3_) as factors. Mean ± SEM values collapsed according to these main factors are provided in SupplementaryTable S3 to facilitate interpretation of the log-transformed plasma biomarker data. n.s = non-significant.

Biomarker concentrations were measured in plasma from WT and CX_3_CR1 KO mice after adolescent stress, alcohol, or combined stress + alcohol exposure. Unless otherwise specified, values were log-transformed prior to three-way ANOVA using genotype, stress, and alcohol as factors. To facilitate interpretation of the log-transformed plasma biomarker data shown in Figure 3, descriptive mean ± SEM values collapsed according to the main factor levels included in the three-way ANOVA model are provided in Supplementary Table S3.

#### 3.2.1 Plasma troponins in WT and CX_3_CR1 KO mice

The analysis revealed a significant main effect of genotype for both cTnI (F_(1,64)_ = 18.06; *p* < 0.001) and cTnT (F_(1,64)_ = 12.90; *p* < 0.001), with higher concentrations in CX_3_CR1 KO mice than in WT mice. No significant main effects of stress or alcohol and no significant interaction terms were detected for either troponin marker (Figures 3A,B).

#### 3.2.2 Plasma CX_3_CL1 in WT and CX_3_CR1 KO mice

For CX_3_CL1, there were significant main effects of genotype (F_(1,65)_ = 858.8; *p* < 0.001) and stress (F_(1,65)_ = 14.04; *p* = 0.001), with no significant main effect of alcohol and no significant interaction terms. CX_3_CR1 KO mice displayed markedly higher CX_3_CL1 concentrations than WT mice, and stress-exposed mice showed higher CX_3_CL1 concentrations than non-stressed mice (Figure 3C).

#### 3.2.3 Plasma ACTH and corticosterone in WT and CX_3_CR1 KO mice

ACTH concentrations showed significant main effects of genotype (F_(1,64)_ = 4.82; *p* = 0.032) and stress (F_(1,64)_ = 4.38; *p* = 0.040), with no significant main effect of alcohol and no significant interaction terms. Across groups, ACTH concentrations were higher in CX_3_CR1 KO mice than in WT mice and were modestly increased in stress-exposed mice compared with non-stressed mice (Figure 3D).

The factorial model for corticosterone revealed significant main effects of genotype (F_(1,64)_ = 5.77; *p* = 0.019), stress (F_(1,64)_ = 14.35; *p* < 0.001), and alcohol (F_(1,64)_ = 9.16; *p* = 0.004), with no significant interaction terms. CX_3_CR1 KO mice displayed lower corticosterone concentrations than WT mice, stress-exposed mice showed lower corticosterone concentrations than non-stressed mice, and alcohol-exposed mice showed lower corticosterone concentrations than saline-treated mice (Figure 3E).

### 3.3 Cardiac gene expression in WT and CX_3_CR1 KO mice

Unless otherwise specified, cardiac gene-expression outcomes were analyzed using three-way ANOVA using genotype, stress, and alcohol as factors.

#### 3.3.1 Chemokines and receptors

Cardiac chemokine-related transcripts showed gene-specific effects of genotype, stress, alcohol, and their interactions. Overall, CX_3_CR1 deficiency was associated with lower expression of several chemokine-related transcripts, whereas adolescent stress and alcohol selectively modulated specific targets.

**FIGURE 4:**
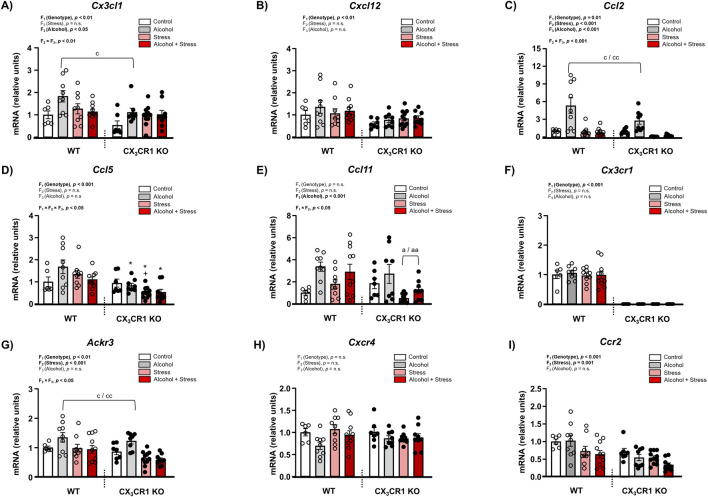
Effects of adolescent stress and/or alcohol exposure on cardiac gene expression of chemokines and their receptors in adult WT and CX_3_CR1 KO mice. **(A)** Relative *Cx3cl1* expression. **(B)** Relative *Cxcl12* expression. **(C)** Relative *Ccl2* expression. **(D)** Relative *Ccl5* expression. **(E)** Relative *Ccl11* expression. **(F)** Relative *Cx3cr1* expression. **(G)** Relative *Ackr3* expression. **(H)** Relative *Cxcr4* expression. **(I)** Relative *Ccr2* expression. Bars represent the mean ± SEM (7–10 mice per group). Data were analyzed using three-way ANOVA with genotype (F_1_), stress (F_2_), and alcohol (F_3_) as factors. *Post hoc* comparisons are shown only when significant: **(A)** indicates significant differences between stress-exposed and non-stressed conditions within the same genotype; (**aa**) indicates significant differences between genotypes under the stress-exposed condition; **(C)** indicates significant differences between the alcohol groups and the control groups, collapsed across genotypes; (**cc**) significant differences between the alcohol groups and the stress + alcohol groups, collapsed across genotypes; (*) indicates significant differences vs. the corresponding group of the other genotype; (+) indicates significant differences vs. the control group in the same genotype. n.s = non-significant.

*Cx3cl1* expression showed significant main effects of genotype (F_(1,62)_ = 9.58; *p* = 0.003) and alcohol (F_(1,62)_ = 6.17; *p* = 0.016), together with a significant stress × alcohol interaction (F_(1,62)_ = 8.04; *p* = 0.006). Consistent with these main effects, CX_3_CR1 KO mice showed lower *Cx3cl1* mRNA levels than WT mice, and alcohol-exposed mice showed higher expression than saline-treated mice. To explore the stress × alcohol interaction, *post hoc* comparisons were performed. Under the no-stress condition, alcohol-treated mice from both genotypes had higher *Cx3cl1* expression than their genotype-matched non-stressed/saline-treated groups (*p* < 0.05). In contrast, no alcohol-related differences were detected under stress exposure in either genotype (Figure 4A).

For *Cxcl12* expression, a significant main effect of genotype was found (F_(1,62)_ = 9.82; *p* = 0.003), with no significant main effects of stress or alcohol and no significant interaction terms. CX_3_CR1 KO mice showed lower *Cxcl12* mRNA levels than WT mice (Figure 4B).

*Ccl2* expression showed significant main effects of genotype (F_(1,62)_ = 7.14; *p* = 0.010), stress (F_(1,62)_ = 26.87; *p* < 0.001), and alcohol (F_(1,62)_ = 16.20; *p* < 0.001), together with a significant stress × alcohol interaction (F_(1,62)_ = 15.30; *p* < 0.001). CX_3_CR1 KO mice showed lower *Ccl2* mRNA levels than WT mice.

Under the no-stress condition, alcohol-treated mice from both genotypes showed higher *Ccl2* expression than their genotype-matched non-stressed/saline-treated groups (*p* < 0.05). This increase was attenuated under stress exposure, as *Ccl2* expression was lower in the stress + alcohol groups than in the corresponding alcohol-treated groups in both genotypes (*p* < 0.05), indicating that the alcohol-related increase observed under no-stress conditions was not maintained when alcohol was combined with stress (Figure 4C).

*Ccl5* expression showed a significant main effect of genotype (F_(1,62)_ = 21.88; *p* < 0.001), together with a significant genotype × stress × alcohol interaction (F_(1,62)_ = 4.38; *p* = 0.041). CX_3_CR1 KO mice showed lower *Ccl5* mRNA levels than WT mice. Under stress exposure, CX_3_CR1 KO mice had lower *Ccl5* expression than WT mice in both the stress and stress + alcohol groups (*p* < 0.05). In addition, within CX_3_CR1 KO mice, stress reduced *Ccl5* expression compared with non-stressed/saline-treated CX_3_CR1 KO mice (*p* < 0.05) (Figure 4D).

*Ccl11* expression was modulated by alcohol and by a genotype-dependent response to stress. A significant main effect of alcohol (F_(1,62)_ = 12.46; *p* < 0.001) and a significant genotype × stress interaction (F_(1,62)_ = 4.92; *p* = 0.030) were observed. Alcohol-exposed mice showed higher *Ccl11* mRNA levels than saline-treated mice. In CX_3_CR1 KO mice, stress reduced *Ccl11* expression under both saline-treated and alcohol-treated conditions compared with the corresponding non-stressed CX_3_CR1 KO groups (*p* < 0.05). Under stress exposure, CX_3_CR1 KO mice also showed lower *Ccl11* expression than WT mice in both the stress and stress + alcohol groups (*p* < 0.05) (Figure 4E).

*Cx3cr1* expression showed a robust main effect of genotype (F_(1,62)_ = 336.3; *p* < 0.001). As expected, *Cx3cr1* expression was virtually absent in CX_3_CR1 KO mice, reflecting the targeted deletion of this gene (Figure 4F).

The factorial analysis for *Ackr3* expression revealed significant main effects of genotype (F_(1,62)_ = 8.34; *p* = 0.005) and stress (F_(1,62)_ = 15.24; *p* < 0.001), together with a significant stress × alcohol interaction (F_(1,62)_ = 5.95; *p* = 0.018). CX_3_CR1 KO mice showed lower *Ackr3* mRNA levels than WT mice, and stress-exposed mice showed lower expression than non-stressed mice. Under the no-stress condition, alcohol-treated mice from both genotypes showed higher *Ackr3* expression than their genotype-matched non-stressed/saline-treated groups (*p* < 0.05). This increase was attenuated under stress exposure, as *Ackr3* expression was lower in the stress + alcohol groups than in the corresponding alcohol-treated groups in both genotypes (*p* < 0.05) (Figure 4G).

Unlike several other chemokine-related transcripts, *Cxcr4* expression was not significantly affected by genotype, stress, alcohol, or their interaction terms (Figure 4H).

For *Ccr2* expression, significant main effects of genotype (F_(1,62)_ = 17.61; *p* < 0.001) and stress (F_(1,62)_ = 11.62; *p* = 0.001) were found. CX_3_CR1 KO mice showed lower *Ccr2* mRNA levels than WT mice, and stress-exposed mice had lower *Ccr2* expression than non-stressed mice (Figure 4I).

#### 3.3.2 Proinflammatory cytokine receptors and NF-κB signaling

**FIGURE 5:**
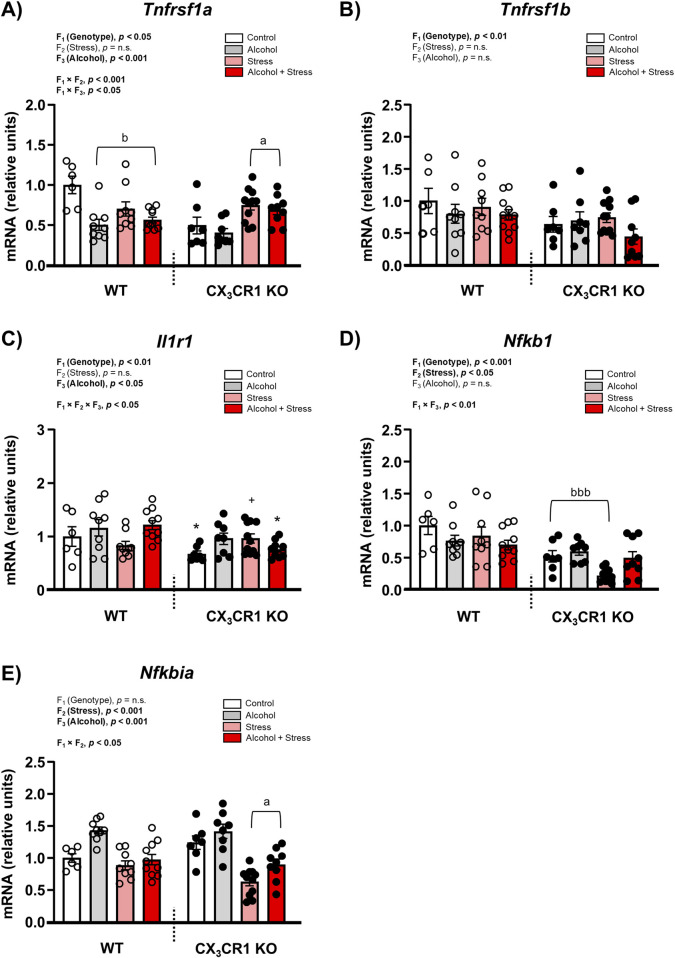
Effects of adolescent stress and/or alcohol exposure on cardiac gene expression of proinflammatory cytokine receptors and NF-κB signaling components in adult WT and CX_3_CR1 KO mice. **(A)** Relative *Tnfrsf1a* expression. **(B)** Relative *Tnfrsf1b* expression. **(C)** Relative *Il1r1* expression. **(D)** Relative *Nfkb1* expression. **(E)** Relative *Nfkbia* expression. Bars represent the mean ± SEM (7–10 mice per group). Data were analyzed using three-way ANOVA with genotype (F_1_), stress (F_2_), and alcohol (F_3_) as factors. *Post hoc* comparisons are shown only when significant: **(A)** indicates significant differences between stress-exposed and non-stressed conditions within the same genotype; **(B)** indicates significant differences between alcohol-treated and saline-treated conditions within the same genotype; (bbb) indicates significant differences between genotypes under the saline-treated condition; (*) indicates significant differences vs. the corresponding group of the other genotype; (+) indicates significant differences vs. the control group in the same genotype. n.s. = non-significant.

*Tnfrsf1a* expression showed significant main effects of genotype (F_(1,62)_ = 4.48; *p* = 0.038) and alcohol (F_(1,62)_ = 14.75; *p* < 0.001), together with significant genotype × stress (F_(1,62)_ = 14.30; *p* < 0.001) and genotype × alcohol (F_(1,62)_ = 5.48; *p* = 0.023) interactions. CX_3_CR1 KO mice showed lower *Tnfrsf1a* mRNA levels than WT mice, and alcohol-exposed mice showed lower expression than saline-treated mice. In CX_3_CR1 KO mice, stress increased *Tnfrsf1a* expression under both saline-treated and alcohol-treated conditions compared with the corresponding non-stressed CX_3_CR1 KO groups (*p* < 0.05). In WT mice, alcohol reduced *Tnfrsf1a* expression under both non-stressed and stressed conditions compared with the corresponding saline-treated WT groups (*p* < 0.05) (Figure 5A).

*Tnfrsf1b* expression was selectively affected by genotype (F_(1,62)_ = 7.78; *p* = 0.007), with CX_3_CR1 KO mice showing lower *Tnfrsf1b* mRNA levels than WT mice (Figure 5B).

*Il1r1* expression showed significant main effects of genotype (F_(1,62)_ = 8.45; *p* = 0.005) and alcohol (F_(1,62)_ = 4.61; *p* = 0.036), together with a significant genotype × stress × alcohol interaction (F_(1,62)_ = 5.78; *p* = 0.019). CX_3_CR1 KO mice showed lower *Il1r1* mRNA levels than WT mice, and alcohol-exposed mice showed higher expression than saline-treated mice. CX_3_CR1 KO mice had lower *Il1r1* expression than WT mice in the non-stressed/saline-treated and stress + alcohol groups (*p* < 0.05). Within CX_3_CR1 KO mice, stress increased *Il1r1* expression under saline-treated conditions compared with non-stressed/saline-treated CX_3_CR1 KO mice (*p* < 0.05) (Figure 5C).

*Nfkb1* expression showed significant main effects of genotype (F_(1,62)_ = 31.18; *p* < 0.001) and stress (F_(1,62)_ = 5.70; *p* = 0.020), together with a significant genotype × alcohol interaction (F_(1,62)_ = 7.57; *p* = 0.008). CX_3_CR1 KO mice showed lower *Nfkb1* mRNA levels than WT mice, and stress-exposed mice showed lower *Nfkb1* expression than non-stressed mice. Under saline-treated conditions, CX_3_CR1 KO mice had lower *Nfkb1* expression than WT mice in both the non-stressed and stress-exposed groups (*p* < 0.05), whereas no genotype-related differences were detected under alcohol-treated conditions (Figure 5D).

For *Nfkbia* expression, significant main effects of stress (F_(1,62)_ = 53.55; *p* < 0.001) and alcohol (F_(1,62)_ = 17.19; *p* < 0.001) were observed, together with a significant genotype × stress interaction (F_(1,62)_ = 5.75; *p* = 0.020). Stress-exposed mice showed lower *Nfkbia* mRNA levels than non-stressed mice, whereas alcohol-exposed mice showed higher expression than saline-treated mice. *Post hoc* comparisons indicated that stress in CX_3_CR1 KO mice reduced *Nfkbia* expression under both saline-treated and alcohol-treated conditions compared with the corresponding non-stressed CX_3_CR1 KO groups (*p* < 0.05) (Figure 5E).

#### 3.3.3 Stress-hormone receptors

**FIGURE 6:**
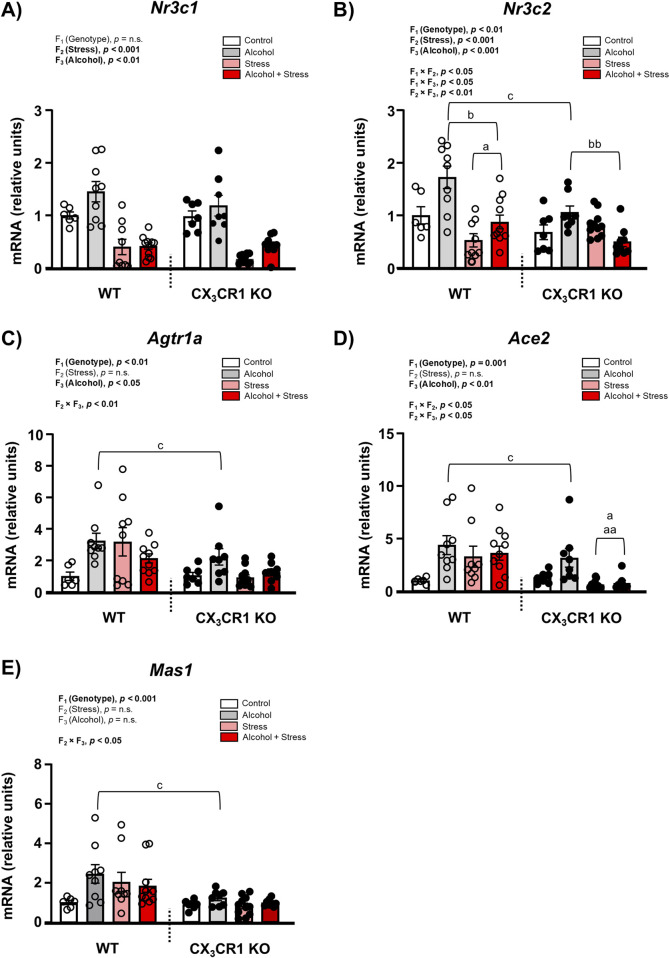
Effects of adolescent stress and/or alcohol exposure on cardiac gene expression of selected glucocorticoid/mineralocorticoid receptor-related and RAAS-related genes in adult WT and CX_3_CR1 KO mice. **(A)** Relative *Nr3c1* expression. **(B)** Relative *Nr3c2* expression. **(C)** Relative *Agtr1a* expression. **(D)** Relative *Ace2* expression. **(E)** Relative *Mas1* expression. Bars represent the mean ± SEM (7–10 mice per group). Data were analyzed using three-way ANOVA with genotype (F_1_), stress (F_2_), and alcohol (F_3_) as factors. *Post hoc* comparisons are shown only when significant: **(A)** indicates significant differences between stress-exposed and non-stressed conditions within the same genotype; (aa) indicates significant differences between genotypes under the stress-exposed condition; **(B)** indicates significant differences between alcohol-treated and saline-treated conditions within the same genotype; **(C)** indicates significant differences between the alcohol groups and the control groups, collapsed across genotypes. n.s = non-significant.

*Nr3c1* expression showed significant main effects of stress (F_(1,62)_ = 86.08; *p* < 0.001) and alcohol (F_(1,62)_ = 7.34; *p* = 0.009). Stress-exposed mice showed lower *Nr3c1* mRNA levels than non-stressed mice, whereas alcohol-exposed mice showed higher *Nr3c1* expression than saline-treated mice (Figure 6A).

For *Nr3c2* expression, the factorial model revealed significant main effects of genotype (F_(1,62)_ = 7.65; *p* = 0.008), stress (F_(1,62)_ = 20.14; *p* < 0.001), and alcohol (F_(1,62)_ = 8.48; *p* = 0.005), together with significant genotype × stress (F_(1,62)_ = 5.53; *p* = 0.022), genotype × alcohol (F_(1,62)_ = 6.88; *p* = 0.011), and stress × alcohol (F_(1,62)_ = 7.69; *p* = 0.007) interactions. CX_3_CR1 KO mice showed lower *Nr3c2* mRNA levels than WT mice, stress-exposed mice showed lower expression than non-stressed mice, and alcohol-exposed mice showed higher expression than saline-treated mice. In WT mice, stress reduced *Nr3c2* expression under both saline-treated and alcohol-treated conditions compared with the corresponding non-stressed WT groups (*p* < 0.05), whereas alcohol increased *Nr3c2* expression under both non-stressed and stress-exposed conditions compared with the corresponding saline-treated WT groups (*p* < 0.05). Under the no-stress condition, alcohol also increased *Nr3c2* expression in CX_3_CR1 KO mice compared with non-stressed/saline-treated CX_3_CR1 KO mice (*p* < 0.05). Genotype differences were evident under alcohol exposure, with CX_3_CR1 KO mice showing lower *Nr3c2* expression than WT mice in both the alcohol and stress + alcohol groups (*p* < 0.05) (Figure 6B).

#### 3.3.4 Renin–angiotensin–aldosterone system (RAAS)-related genes

*Agtr1a* expression showed significant main effects of genotype (F_(1,61)_ = 9.62; *p* = 0.003) and alcohol (F_(1,61)_ = 4.11; *p* = 0.047), together with a significant stress × alcohol interaction (F_(1,61)_ = 9.80; *p* = 0.003). CX_3_CR1 KO mice showed lower *Agtr1a* mRNA levels than WT mice, and alcohol-exposed mice showed higher expression than saline-treated mice. Under the no-stress condition, alcohol-treated mice from both genotypes had higher *Agtr1a* expression than their genotype-matched non-stressed/saline-treated groups (*p* < 0.05). No significant exposure-related differences were detected under stress exposure (Figure 6C).

*Ace2* expression showed significant main effects of genotype (F_(1,61)_ = 11.32; *p* = 0.001) and alcohol (F_(1,61)_ = 8.94; *p* = 0.004), together with significant genotype × stress (F_(1,61)_ = 5.72; *p* = 0.020) and stress × alcohol (F_(1,61)_ = 6.22; *p* = 0.015) interactions. CX_3_CR1 KO mice showed lower *Ace2* mRNA levels than WT mice, and alcohol-exposed mice showed higher expression than saline-treated mice. Under the no-stress condition, alcohol-treated mice from both genotypes had higher *Ace2* expression than their genotype-matched non-stressed/saline-treated groups (*p* < 0.05). In CX_3_CR1 KO mice, stress reduced *Ace2* expression under both saline-treated and alcohol-treated conditions compared with the corresponding non-stressed CX_3_CR1 KO groups (*p* < 0.05). Under stress exposure, CX_3_CR1 KO mice also showed lower *Ace2* expression than WT mice in both the stress and stress + alcohol groups (*p* < 0.05) (Figure 6D).

For *Mas1* expression, a significant main effect of genotype (F_(1,61)_ = 14.76; *p* < 0.001) was observed, together with a significant stress × alcohol interaction (F_(1,61)_ = 4.13; *p* = 0.047). CX_3_CR1 KO mice showed lower *Mas1* mRNA levels than WT mice. Under the no-stress condition, alcohol-treated mice from both genotypes had higher *Mas1* expression than their genotype-matched non-stressed/saline-treated groups (*p* < 0.05). No significant alcohol-related differences were detected under stress exposure (Figure 6E).

#### 3.3.5 Targeted cardiac stress and remodeling markers

To complement the troponin and main cardiac transcriptional findings, we performed a targeted qPCR analysis of selected cardiac stress/remodeling markers, including *Nppa*, *Nppb*, and *Myh7*. Given its complementary purpose and different qPCR methodology, this analysis is reported separately in Supplementary Figure S3.

Three-way ANOVA for *Nppa* expression revealed a significant main effect of genotype (F_(1,56)_ = 23.91; *p* < 0.001) and a significant genotype × alcohol interaction (F_(1,56)_ = 8.92; *p* = 0.004). Overall, CX_3_CR1 KO mice showed higher *Nppa* mRNA levels than WT mice. Alcohol reduced *Nppa* expression in WT mice under the no-stress condition. In addition, CX_3_CR1 KO mice had higher *Nppa* expression than WT mice in both alcohol-treated conditions, namely alcohol and stress + alcohol groups (Supplementary Figure S3A).

*Nppb* expression showed significant main effects of stress (F_(1,59)_ = 9.26; *p* = 0.004) and alcohol (F_(1,59)_ = 15.43; *p* < 0.001), together with significant stress × alcohol (F_(1,59)_ = 6.87; *p* = 0.011) and genotype × stress × alcohol (F_(1,59)_ = 4.72; *p* = 0.034) interactions. Alcohol increased *Nppb* expression under the no-stress condition, particularly in CX_3_CR1 KO mice. In WT mice, combined stress + alcohol exposure increased *Nppb* expression compared with non-stressed/saline-treated WT mice, and this WT stress + alcohol group also showed higher *Nppb* expression than the corresponding CX_3_CR1 KO stress + alcohol group (Supplementary Figure S3B).

*Myh7* expression showed significant main effects of stress (F_(1,60)_ = 5.36; *p* = 0.024) and alcohol (F_(1,60)_ = 17.52; *p* < 0.001). Alcohol-exposed mice showed higher *Myh7* mRNA levels than saline-treated mice, whereas stress-exposed mice showed lower *Myh7* expression than non-stressed mice (Supplementary Figure S3C).

Together, this complementary analysis suggests selective modulation of cardiac stress/remodeling-related markers alongside the troponin and inflammatory/RAAS-related transcriptional findings. *Nppa* and *Nppb* provided the most relevant molecular context for the circulating troponin findings, whereas *Myh7* appeared to reflect exposure-related remodeling responses rather than genotype-dependent cardiac injury biomarker changes. Because these markers were assessed using a different qPCR approach, they were interpreted as supportive molecular evidence rather than as part of the primary cardiac gene-expression panel.

### 3.4 Association between plasma biomarkers and cardiac gene expression in WT and CX_3_CR1 KO mice

**FIGURE 7:**
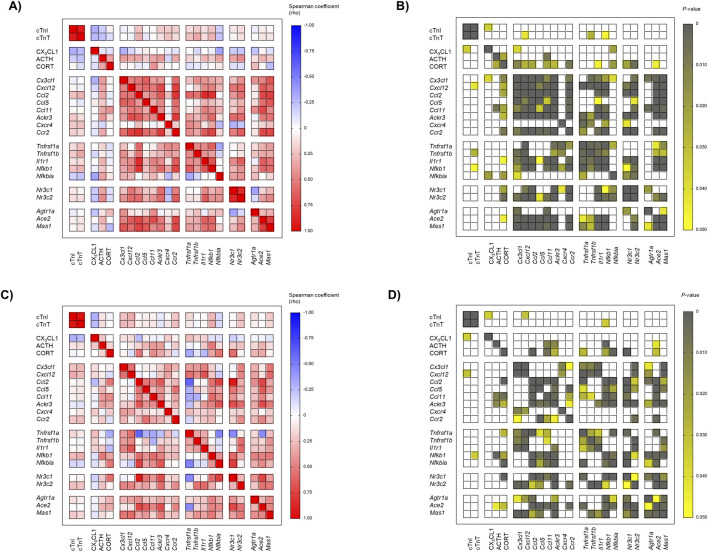
Correlation and significance maps between plasma biomarkers and cardiac gene expression in adult WT and CX_3_CR1 KO mice. Spearman correlation analyses were performed separately in WT and CX_3_CR1 KO mice, pooling all exposure conditions within each genotype. **(A)** Full Spearman correlation coefficients between plasma biomarkers and cardiac gene-expression markers in WT mice. **(B)** Corresponding significance map highlighting statistically significant correlations in WT mice. **(C)** Full Spearman correlation coefficients between plasma biomarkers and cardiac gene-expression markers in CX_3_CR1 KO mice. **(D)** Corresponding significance map highlighting statistically significant correlations in CX_3_CR1 KO mice. Correlation heatmaps represent Spearman’s rho values from −1 to 1, whereas significance maps display the corresponding statistically significant *p*-values (*p* < 0.05). Panels **(B,D)** are derived from the same correlation analyses shown in panels **(A,C)** and are included to facilitate visual identification of significant associations. Correlations are exploratory and pooled across exposure groups within each genotype.

Correlation analyses were conducted separately within WT and CX_3_CR1 KO mice to examine potential associations between circulating biomarkers and cardiac mRNA levels (Figure 7).

In WT mice (Figures 7A,B), plasma troponins were tightly coupled (cTnI–cTnT: rho = 0.92, *p* < 0.001) and cTnI correlated negatively with plasma CX_3_CL1 (rho = −0.31, *p* < 0.05). Several troponin–gene links were evident, such as cTnT with *Tnfrsf1b* (rho = 0.33, *p* < 0.05). The heatmaps also showed a prominent chemokine-centered module: *Cxcl12* correlated strongly with *Ackr3* (rho = 0.81, *p* < 0.001), *Ace2* (rho = 0.72, *p* < 0.001), and *Ccr2* (rho = 0.65, *p* < 0.001). Robust associations were observed among proinflammatory receptors and NF-κB signaling, including *Il1r1*–*Nfkb1* (rho = 0.67, *p* < 0.001) and *Tnfrsf1a*–*Nfkb1* (rho = 0.63, *p* < 0.001). Plasma CX_3_CL1 correlated negatively with *Nfkbia* (rho = −0.35, *p* < 0.05) and showed a negative association with cardiac *Cx3cl1* mRNA (rho = −0.31, *p* < 0.05). ACTH and CORT also displayed selective links, such as CORT–*Ccr2* (rho = 0.41, *p* < 0.01).

In CX_3_CR1 KO mice (Figures 7C,D), the overall network was attenuated and partially reorganized. The strong cTnI–cTnT correlation persisted (rho = 0.95, *p* < 0.001) and the cTnI–CX_3_CL1 association remained negative (rho = −0.31, *p* < 0.05), but several WT patterns were lost (e.g., cTnT–*Tnfrsf1b* became non-significant; the *Cxcl12* module with *Ackr3* and *Ace2* no longer reached significance). Conversely, CX_3_CR1 KO mice gained distinct couplings linking stress-hormone receptors, NF-κB regulation, and the renin–angiotensin–Mas axis: *Nr3c1*–*Mas1* (rho = −0.75, *p* < 0.001), *Nfkbia*–*Mas1* (rho = −0.73, *p* < 0.001), *Nfkbia*–*Ace2* (rho = 0.45, *p* < 0.01), and *Ackr3*–*Ace2* (rho = 0.66, *p* < 0.001); ACTH also correlated with *Nfkb1* (rho = 0.52, *p* < 0.001).

Two relationships reversed direction and were significant in both genotypes: *Tnfrsf1a*–*Ace2* (WT mice: rho = 0.29, *p* < 0.05; and CX_3_CR1 KO: rho = −0.46, *p* < 0.01) and *Nr3c1*–*Agtr1a* (WT mice: rho = −0.36, *p* < 0.05; and CX_3_CR1 KO: rho = 0.41, *p* < 0.01).

Taken together, Figure 7 indicates that WT mice display a dense chemokine/*Cxcl12*-anchored architecture integrating inflammatory and RAAS-related transcripts, whereas CX_3_CR1 deficiency reduces this coordination and shifts the association structure toward links among glucocorticoid/mineralocorticoid receptor-related genes, NF-κB/IκBα signaling, and ACE2/Mas-related transcripts. These exploratory correlation patterns complement the three-way ANOVA results and should be interpreted as hypothesis-generating.

## 4 Discussion

The present study was designed to examine whether CX_3_CR1 signaling participates in the long-term coordination of behavioral, endocrine, and cardiac molecular responses after adolescent stress and alcohol exposure. Rather than representing two unrelated domains, anxiety-like behavior and endocrine–cardiac signatures were interpreted as complementary readouts of long-term responses to adolescent stress and alcohol exposure. In this framework, EPM behavior reflects adult stress-related behavioral output, ACTH and corticosterone reflect HPA-axis activity, circulating cTnI/cTnT reflect cardiac injury biomarker release, plasma CX_3_CL1 reflects systemic fractalkine signaling, and cardiac inflammatory/RAAS-related transcripts and targeted stress/remodeling markers provide indirect molecular evidence of cardiac biomarker and transcriptional vulnerability.

This study shows that CX_3_CR1 deficiency is associated with a distinct adult neuroendocrine–cardiac phenotype and with selective exposure-dependent changes in specific inflammatory and receptor-related genes, rather than a uniform amplification of all stress/alcohol effects. Behaviorally, CX_3_CR1 KO mice displayed a higher anxiety index and reduced open-arm exploration under selected exposure conditions, and the CX_3_CR1 antagonist AZD8797 partially reproduced these anxiety-like behavioral effects in WT mice. Systemically, CX_3_CR1 KO mice showed higher plasma cTnI/cTnT and CX_3_CL1, elevated ACTH, and lower corticosterone, while stress and alcohol exposure were also associated with lower corticosterone. In cardiac tissue, alcohol exposure, mainly under the no-stress condition, increased *Cx3cl1, Ccl2*, *Ackr3* and selected RAAS-related transcripts, whereas genotype effects were characterized by broadly lower expression across chemokine, NF-κB-related, mineralocorticoid receptor-related, and RAAS-related targets. Exploratory correlation patterns further suggested that CX_3_CR1 deficiency weakens the association structure among chemokine-related transcripts and shifts the pattern of correlations toward links among glucocorticoid/mineralocorticoid receptor-related genes, NF-κB/IκBα signaling, and RAAS-related transcripts. A targeted supplementary qPCR analysis further indicated selective modulation of cardiac stress/remodeling markers. Among these, *Nppa* and *Nppb* provided complementary natriuretic peptide-related molecular context for the circulating troponin findings, whereas *Myh7* reflected additional exposure-related remodeling responses. These data provide supportive molecular information alongside the inflammatory/RAAS-related transcriptional findings, but do not replace direct functional or histopathological validation.

The CX_3_CL1/CX_3_CR1 axis mediates signaling between neurons and microglia and contributes to the regulation of microglial activation, synaptic remodeling, and neuroimmune responses. Disrupting CX_3_CR1 increases microglial reactivity and vulnerability to stress-related behavioral phenotypes, consistent with the anxiety-like behavior and endocrine dysregulation observed here. Cardona et al. demonstrated that CX_3_CR1 signaling restrains microglial neurotoxicity *in vivo* (Cardona et al., 2006), and subsequent work has consolidated its role in neuron–glia communication and stress responsivity (Sheridan and Murphy, 2013). Previous studies have linked CX_3_CL1/CX_3_CR1 signaling to stress-related neuroendocrine regulation. Winkler et al. showed that impaired microglial fractalkine signaling alters stress-related behavioral responses and HPA-axis reactivity to acute stress in mice, including ACTH and corticosterone-related outcomes (Winkler et al., 2017). In our previous work, CX_3_CR1-deficient mice exposed to adolescent stress and alcohol displayed altered hypothalamic CRH-pathway gene expression, attenuated corticosterone responses to stress, and loss of the normal ACTH–corticosterone correlation (Medina-Vera et al., 2025). More recently, prenatal–lactational alcohol exposure was shown to induce sex-specific CX_3_CL1/CX_3_CR1 dysregulation linked to neuroendocrine imbalance and cardiovascular risk, further supporting the relevance of this pathway in long-term neuroendocrine–cardiovascular programming after early-life alcohol exposure (Medina-Vera et al., 2026). In addition, chronic social instability stress has been reported to upregulate brain *Cx3cr1* expression and alter neuroimmune markers in female mice, although corticosterone levels were not significantly changed (Díez-Solinska et al., 2022).

Adolescence is a sensitive developmental window in which stress and alcohol can produce persistent HPA-axis and neuroimmune alterations into adulthood. The pattern of higher ACTH with lower corticosterone in CX_3_CR1 KO mice suggests altered pituitary-adrenal coupling, in which increased upstream ACTH drive is not matched by a corresponding adrenal corticosterone response. This endocrine dissociation is consistent with altered HPA feedforward signaling and/or reduced adrenal responsiveness rather than direct evidence of altered feedback regulation. The exposure-related regulation of cardiac *Nr3c1*, together with the genotype- and exposure-dependent modulation of *Nr3c2*, further supports long-term disruption of glucocorticoid/mineralocorticoid receptor-related signaling. These results extend our prior findings showing altered stress-related behavioral responses, disrupted hypothalamic stress-related signaling, altered HPA-axis regulation, and exaggerated systemic inflammation after adolescent stress/alcohol exposure in CX_3_CR1-deficient mice (Medina-Vera et al., 2025).

Beyond the CNS, the CX_3_CL1/CX_3_CR1 axis contributes to cardiovascular pathology (Stangret et al., 2024; Iwahashi et al., 2025). In human STEMI, elevated fractalkine is associated with worse tissue-level outcomes and prognosis (Boag et al., 2015; Xu et al., 2019), and CX_3_CR1-dependent lymphocyte dynamics have been linked to microvascular obstruction and adverse remodeling after reperfusion (Boag et al., 2015; Spray et al., 2021). Our observation of higher troponin levels in CX_3_CR1 KO mice, together with broad dampening of cardiac inflammatory transcripts, suggests that fractalkine signaling may influence the balance between injury markers and compensatory inflammatory responses rather than acting as a purely pro-injury pathway (Gu et al., 2015; Iwahashi et al., 2025).

Preclinical and clinical data support therapeutic targeting of CX_3_CR1 in ischemic heart disease. Neutralization of fractalkine post-myocardial infarction improves function in mice, and selective CX_3_CR1 blockade is under clinical evaluation for limiting inflammatory injury after myocardial infarction (D’Haese et al., 2012; Loh et al., 2023). While our model is not an infarction paradigm, the AZD8797 behavioral validation supports the contribution of acute CX_3_CR1 antagonism to anxiety-like behavioral outcomes. However, because plasma and cardiac samples were not collected from this cohort, the pharmacological experiment cannot be used to infer direct endocrine or cardiac molecular effects.

At the cardiac transcriptional level, the factorial models and exploratory correlations together suggest that CX_3_CR1 contributes to organizing coordinated chemokine/TNF–NF-κB–RAAS transcriptional relationships in WT hearts. Alcohol-related increases in *Cx3cl1*, *Ccl2*, and *Ackr3* were most evident under the no-stress condition, and WT correlation matrices indicated strong coupling between *Cxcl12* and *Ackr3*, inflammatory receptors (*Tnfrsf1a/b*, *Il1r1*), NF-κB signaling (*Nfkb1*), and RAAS-related genes such as *Ace2*. In CX_3_CR1 KO hearts, this architecture was attenuated and partially re-routed toward glucocorticoid/mineralocorticoid receptor–NF-κB/IκBα–RAAS couplings, consistent with compensatory reliance on steroid-receptor/NF-κB checkpoints to interface with the ACE2/Ang-(1–7)/Mas axis (Simões e Silva et al., 2013; Rodrigues Prestes et al., 2017; Santos et al., 2018). This interpretation is biologically plausible given the anti-inflammatory and NF-κB-suppressive properties of ACE2/Ang-(1–7)/Mas signaling (Li et al., 2015; Villalobos et al., 2016).

Importantly, genotype-by-exposure effects were not uniformly expressed across endpoints; instead, they clustered in selected inflammatory and steroid-receptor nodes (e.g., *Tnfrsf1a*, *Il1r1*, *Nfkb1*, *Nfkbia*, *Nr3c2*, and *Ace2*) and in EPM open-arm behavior. Together, these findings suggest that the contribution of CX_3_CR1 to endocrine–cardiac molecular responses depends on the specific adolescent exposure condition, rather than reflecting a uniform amplification of stress or alcohol effects. The network-level correlation structure should be interpreted as hypothesis-generating.

### 4.1 Limitations

First, behavioral assessment relied primarily on the EPM; incorporating orthogonal assays, such as the open-field and light–dark box tests, would strengthen the behavioral inference. Second, groups were balanced by sex, whereas analyses were not designed or powered to yield sex-stratified conclusions; given evidence for sex-specific fractalkine signaling and potential differences in HPA- and RAAS-related pathways, future studies should explicitly test sex effects in a dedicated design. Third, repeated i.p. alcohol administration may add an aversive, handling-related stress component beyond ethanol pharmacology; while saline injections controlled for handling and injection exposure, future work should directly compare forced administration with voluntary drinking paradigms to disentangle these contributions. Fourth, plasma biomarkers and cardiac mRNA were assessed at a single post-exposure time point; therefore, longitudinal sampling, time-course analyses, and protein-level validation, including cellular localization, are needed to confirm temporal dynamics and mechanism. Although we added a targeted supplementary qPCR analysis of cardiac stress/remodeling-related markers (*Nppa*, *Nppb*, and *Myh7*) to provide molecular context for circulating troponins and cardiac transcriptional profiling, this molecular assessment does not replace direct histopathological or functional validation. Therefore, we cannot determine whether these biomarker and transcriptional changes reflect overt myocardial injury, inflammatory infiltration, fibrosis, apoptosis, tissue remodeling, or altered cardiac performance. Future studies should combine circulating biomarkers and cardiac mRNA profiling with echocardiography or cardiac MRI, as well as histological assessment of inflammatory cell infiltration, fibrosis, cardiomyocyte injury, and tissue remodeling. Fifth, the *Cx3cr1* ^GFP^ knock-in/knock-out background may entail developmental compensation. Finally, correlation analyses were exploratory and not adjusted for multiplicity across all tested nodes; thus, network-level inferences should be validated in independent cohorts or prospectively designed experiments.

### 4.2 Conclusions

These findings suggest that CX_3_CR1 signaling contributes to the long-term coordination of behavioral stress-related responses, HPA-axis activity, circulating cardiac injury biomarkers, and cardiac inflammatory/RAAS-related transcriptional programs after adolescent stress and alcohol exposure. Disruption of this pathway was associated with increased anxiety-like behavior, altered neuroendocrine output, elevated circulating cardiac injury biomarkers, and reconfiguration of cardiac inflammatory–neuroendocrine–RAAS-related transcriptional associations. In WT hearts, fractalkine signaling aligned with coordinated chemokine/TNF–NF-κB–RAAS transcriptional patterns, whereas CX_3_CR1 deficiency attenuated this coordination and shifted the association structure toward links among glucocorticoid/mineralocorticoid receptor-related genes, NF-κB/IκBα signaling, and ACE2/Mas-related transcripts. Together, these results support a modulatory role for CX_3_CR1 in linking adolescent stress/alcohol exposure with persistent neuroendocrine and cardiac molecular vulnerability. Because cardiac function and histopathology were not assessed, these results should be interpreted as evidence of cardiac biomarker and molecular vulnerability rather than definitive myocardial pathology.

## Data Availability

The datasets presented in this study can be found in online repositories. The names of the repository/repositories and accession number(s) can be found below: https://doi.org/10.5281/zenodo.17661546.

## References

[B1] Anderson R. I. Lopez M. F. Becker H. C. (2016). Stress-induced enhancement of ethanol intake in C57BL/6J mice with a history of chronic ethanol exposure: involvement of kappa opioid receptors. Front. Cell. Neurosci. 10, 45. 10.3389/fncel.2016.00045 26941607 PMC4763044

[B2] Arora M. ElSayed A. Beger B. Naidoo P. Shilton T. Jain N. (2022). The impact of alcohol consumption on cardiovascular health: myths and measures. Glob. Heart 17, 45. 10.5334/gh.1132 36051324 PMC9306675

[B3] Balan I. Grusca A. Chéry S. L. Materia B. R. O’Buckley T. K. Morrow A. L. (2024). Neurosteroid [3α,5α]-3-Hydroxy-pregnan-20-one enhances the CX3CL1-CX3CR1 pathway in the brain of alcohol-preferring rats with sex-specificity. Life 14, 860. 10.3390/life14070860 39063614 PMC11277648

[B4] Bazan J. F. Bacon K. B. Hardiman G. Wang W. Soo K. Rossi D. (1997). A new class of membrane-bound chemokine with a CX3C motif. Nature 385, 640–644. 10.1038/385640a0 9024663

[B5] Boag S. E. Das R. Shmeleva E. V. Bagnall A. Egred M. Howard N. (2015). T lymphocytes and fractalkine contribute to myocardial ischemia/reperfusion injury in patients. J. Clin. Invest. 125, 3063–3076. 10.1172/JCI80055 26168217 PMC4563749

[B6] Boden J. M. Fergusson D. M. (2011). Alcohol and depression. Addict. Abingdon Engl. 106, 906–914. 10.1111/j.1360-0443.2010.03351.x 21382111

[B7] Cardona A. E. Pioro E. P. Sasse M. E. Kostenko V. Cardona S. M. Dijkstra I. M. (2006). Control of microglial neurotoxicity by the fractalkine receptor. Nat. Neurosci. 9, 917–924. 10.1038/nn1715 16732273

[B8] Cederblad L. Rosengren B. Ryberg E. Hermansson N.-O. (2016). AZD8797 is an allosteric non-competitive modulator of the human CX3CR1 receptor. Biochem. J. 473, 641–649. 10.1042/BJ20150520 26656484 PMC4764977

[B9] Combadière C. Potteaux S. Gao J.-L. Esposito B. Casanova S. Lee E. J. (2003). Decreased atherosclerotic lesion formation in CX3CR1/apolipoprotein E double knockout mice. Circulation 107, 1009–1016. 10.1161/01.cir.0000057548.68243.42 12600915

[B10] Crews F. T. Sarkar D. K. Qin L. Zou J. Boyadjieva N. Vetreno R. P. (2015). Neuroimmune function and the consequences of alcohol exposure. Alcohol Res. Curr. Rev. 37 (331–341), 344–351. 10.35946/arcr.v37.2.15 26695754 PMC4590627

[B11] Damås J. K. Boullier A. Wæhre T. Smith C. Sandberg W. J. Green S. (2005). Expression of fractalkine (CX3CL1) and its receptor, CX3CR1, is elevated in coronary artery disease and is reduced during statin therapy. Arterioscler. Thromb. Vasc. Biol. 25, 2567–2572. 10.1161/01.ATV.0000190672.36490.7b 16224053

[B12] Díez-Solinska A. Lebeña A. Garmendia L. Labaka A. Azkona G. Perez-Tejada J. (2022). Chronic social instability stress down-regulates IL-10 and up-regulates CX3CR1 in tumor-bearing and non-tumor-bearing female mice. Behav. Brain Res. 435, 114063. 10.1016/j.bbr.2022.114063 35988637

[B13] Doremus-Fitzwater T. L. Deak T. (2022). Adolescent neuroimmune function and its interaction with alcohol. Int. Rev. Neurobiol. 161, 167–208. 10.1016/bs.irn.2021.08.006 34801169 PMC9204461

[B14] D’Haese J. G. Friess H. Ceyhan G. O. (2012). Therapeutic potential of the chemokine–receptor duo fractalkine/CX3CR1: an update. Expert Opin. Ther. Targets 16, 613–618. 10.1517/14728222.2012.682574 22530606

[B15] Eiland L. Romeo R. D. (2013). Stress and the developing adolescent brain. Neuroscience 249, 162–171. 10.1016/j.neuroscience.2012.10.048 23123920 PMC3601560

[B16] Gu X. Xu J. Yang X.-P. Peterson E. Harding P. (2015). Fractalkine neutralization improves cardiac function after myocardial infarction. Exp. Physiol. 100, 805–817. 10.1113/EP085104 25943588 PMC4686137

[B17] Hellwig S. Brioschi S. Dieni S. Frings L. Masuch A. Blank T. (2016). Altered microglia morphology and higher resilience to stress-induced depression-like behavior in CX3CR1-deficient mice. Brain. Behav. Immun. 55, 126–137. 10.1016/j.bbi.2015.11.008 26576722

[B18] Imai T. Hieshima K. Haskell C. Baba M. Nagira M. Nishimura M. (1997). Identification and molecular characterization of fractalkine receptor CX3CR1, which mediates both leukocyte migration and adhesion. Cell 91, 521–530. 10.1016/s0092-8674(00)80438-9 9390561

[B19] Iwahashi Y. Ishida Y. Mukaida N. Kondo T. Iwahashi Y. (2025). Pathophysiological roles of the CX3CL1-CX3CR1 axis in renal disease, cardiovascular disease, and cancer. Int. J. Mol. Sci. 26, 5352. 10.3390/ijms26115352 40508161 PMC12155443

[B20] Jiménez-López R. Romero-Trevejo J. L. Fernández-Romero L. Martín-Chaves L. Romero-Cuevas M. Molina-Ramos A. I. (2024). Differential ophthalmological profile in patients with coronary artery disease coexisting with type 2 diabetes mellitus: elevated tear cytokine concentrations. J. Clin. Med. 13, 4906. 10.3390/jcm13164906 39201047 PMC11355890

[B21] Jung S. Aliberti J. Graemmel P. Sunshine M. J. Kreutzberg G. W. Sher A. (2000). Analysis of fractalkine receptor CX(3)CR1 function by targeted deletion and green fluorescent protein reporter gene insertion. Mol. Cell. Biol. 20, 4106–4114. 10.1128/MCB.20.11.4106-4114.2000 10805752 PMC85780

[B22] Kilkenny C. Browne W. J. Cuthill I. C. Emerson M. Altman D. G. (2010). Improving bioscience research reporting: the ARRIVE guidelines for reporting animal research. PLoS Biol. 8, e1000412. 10.1371/journal.pbio.1000412 20613859 PMC2893951

[B23] Li Y. Cao Y. Zeng Z. Liang M. Xue Y. Xi C. (2015). Angiotensin-converting enzyme 2/angiotensin-(1-7)/Mas axis prevents lipopolysaccharide-induced apoptosis of pulmonary microvascular endothelial cells by inhibiting JNK/NF-κB pathways. Sci. Rep. 5, 8209. 10.1038/srep08209 25644821 PMC4314638

[B24] Loh S. X. Ekinci Y. Spray L. Jeyalan V. Olin T. Richardson G. (2023). Fractalkine signalling (CX3CL1/CX3CR1 axis) as an emerging target in coronary artery disease. J. Clin. Med. 12, 4821. 10.3390/jcm12144821 37510939 PMC10381654

[B25] Medina-Vera D. Martín-Chaves L. Sánchez-Marín L. Díaz-Ottaviano M. Gavito A. L. Popova O. (2025). Maladaptive stress-coping behavior in CX3CR1-Deficient mice: impact of adolescent stress and alcohol exposure on neuroimmune responses and inflammation. Neuropharmacology 275, 110503. 10.1016/j.neuropharm.2025.110503 40339639

[B26] Medina-Vera D. García-Baos A. Medrano M. Martín-Chaves L. Rodríguez-Capitán J. Rodríguez de Fonseca F. (2026). Prenatal-lactational alcohol exposure induces sex-specific CX3CL1/CX3CR1 dysregulation linked to neuroendocrine imbalance and cardiovascular risk. Brain. Behav. Immun. 134, 106463. 10.1016/j.bbi.2026.106463 41605307

[B27] Milior G. Lecours C. Samson L. Bisht K. Poggini S. Pagani F. (2016). Fractalkine receptor deficiency impairs microglial and neuronal responsiveness to chronic stress. Brain. Behav. Immun. 55, 114–125. 10.1016/j.bbi.2015.07.024 26231972

[B28] Paolicelli R. C. Bolasco G. Pagani F. Maggi L. Scianni M. Panzanelli P. (2011). Synaptic pruning by microglia is necessary for normal brain development. Science 333, 1456–1458. 10.1126/science.1202529 21778362

[B29] Paolicelli R. C. Bisht K. Tremblay M.-È. (2014). Fractalkine regulation of microglial physiology and consequences on the brain and behavior. Front. Cell. Neurosci. 8, 129. 10.3389/fncel.2014.00129 24860431 PMC4026677

[B30] Piano M. R. (2017). Alcohol’s effects on the cardiovascular system. Alcohol Res. Curr. Rev. 38, 219–241. 10.35946/arcr.v38.2.06 28988575 PMC5513687

[B31] Porras-Perales Ó. Segovia-Reyes J. Crespo-Delgado Á. Ruiz-González D. Flores-López M. Medina-Vera D. (2025). Distinct cardiac troponin alterations in patients with cocaine and alcohol use disorders during abstinence for cardiovascular risk assessment. Sci. Rep. 15, 21887. 10.1038/s41598-025-08041-y 40594766 PMC12214730

[B32] Richter B. Koller L. Hohensinner P. J. Rychli K. Zorn G. Goliasch G. (2012). Fractalkine is an independent predictor of mortality in patients with advanced heart failure. Thromb. Haemost. 108, 1220–1227. 10.1160/TH12-03-0195 23014777

[B33] Rodrigues Prestes T. R. Rocha N. P. Miranda A. S. Teixeira A. L. Simoes-E-Silva A. C. (2017). The anti-inflammatory potential of ACE2/Angiotensin-(1-7)/Mas receptor axis: evidence from basic and clinical research. Curr. Drug Targets 18, 1301–1313. 10.2174/1389450117666160727142401 27469342

[B34] Sánchez-Marín L. Flores-López M. Gavito A. L. Suárez J. Pavón-Morón F. J. de Fonseca F. R. (2022a). Repeated restraint stress and binge alcohol during adolescence induce long-term effects on anxiety-like behavior and the expression of the endocannabinoid system in Male rats. Biomedicines 10, 593. 10.3390/biomedicines10030593 35327395 PMC8945821

[B35] Sánchez-Marín L. Flores-López M. Pastor A. Gavito A. L. Suárez J. de la Torre R. (2022b). Acute stress and alcohol exposure during adolescence result in an anxious phenotype in adulthood: role of altered glutamate/endocannabinoid transmission mechanisms. Prog. Neuropsychopharmacol. Biol. Psychiatry 113, 110460. 10.1016/j.pnpbp.2021.110460 34695542

[B36] Santos R. A. S. Sampaio W. O. Alzamora A. C. Motta-Santos D. Alenina N. Bader M. (2018). The ACE2/Angiotensin-(1-7)/MAS axis of the renin-angiotensin system: focus on Angiotensin-(1-7). Physiol. Rev. 98, 505–553. 10.1152/physrev.00023.2016 29351514 PMC7203574

[B37] Shah R. Matthews G. J. Shah R. Y. McLaughlin C. Chen J. Wolman M. (2015). Serum fractalkine (CX3CL1) and cardiovascular outcomes and diabetes: findings from the chronic renal insufficiency cohort (CRIC) study. Am. J. Kidney Dis. Off. J. Natl. Kidney Found. 66, 266–273. 10.1053/j.ajkd.2015.01.021 25795074 PMC4516570

[B38] Sheridan G. K. Murphy K. J. (2013). Neuron-glia crosstalk in health and disease: fractalkine and CX3CR1 take centre stage. Open Biol. 3, 130181. 10.1098/rsob.130181 24352739 PMC3877844

[B39] Simões e Silva A. Silveira K. Ferreira A. Teixeira M. (2013). ACE2, angiotensin-(1-7) and mas receptor axis in inflammation and fibrosis. Br. J. Pharmacol. 169, 477–492. 10.1111/bph.12159 23488800 PMC3682698

[B40] Spray L. Park C. Cormack S. Mohammed A. Panahi P. Boag S. (2021). The fractalkine receptor CX3CR1 links lymphocyte kinetics in CMV-seropositive patients and acute myocardial infarction with adverse left ventricular remodeling. Front. Immunol. 12, 605857. 10.3389/fimmu.2021.605857 34046028 PMC8147691

[B41] Stangret A. Sadowski K. A. Jabłoński K. Kochman J. Opolski G. Grabowski M. (2024). Chemokine fractalkine and non-obstructive coronary artery disease—Is there a link? Int. J. Mol. Sci. 25, 3885. 10.3390/ijms25073885 38612695 PMC11012077

[B42] Teupser D. Pavlides S. Tan M. Gutierrez-Ramos J.-C. Kolbeck R. Breslow J. L. (2004). Major reduction of atherosclerosis in fractalkine (CX3CL1)-deficient mice is at the brachiocephalic artery, not the aortic root. Proc. Natl. Acad. Sci. 101, 17795–17800. 10.1073/pnas.0408096101 15596719 PMC539720

[B43] Tottenham N. Galván A. (2016). Stress and the adolescent brain: amygdala-prefrontal cortex circuitry and ventral striatum as developmental targets. Neurosci. Biobehav. Rev. 70, 217–227. 10.1016/j.neubiorev.2016.07.030 27473936 PMC5074883

[B44] Villalobos L. A. San Hipólito-Luengo Á. Ramos-González M. Cercas E. Vallejo S. Romero A. (2016). The Angiotensin-(1-7)/Mas axis counteracts angiotensin II-Dependent and -Independent pro-inflammatory signaling in human vascular smooth muscle cells. Front. Pharmacol. 7, 482. 10.3389/fphar.2016.00482 28018220 PMC5156706

[B45] Walter T. J. Vetreno R. P. Crews F. T. (2017). Alcohol and stress activation of microglia and neurons: brain regional effects. Alcohol. Clin. Exp. Res. 41, 2066–2081. 10.1111/acer.13511 28941277 PMC5725687

[B46] Whitcomb B. W. Schisterman E. F. (2008). Assays with lower detection limits: implications for epidemiological investigations. Paediatr. Perinat. Epidemiol. 22, 597–602. 10.1111/j.1365-3016.2008.00969.x 19000298 PMC2723785

[B47] Winkler Z. Kuti D. Ferenczi S. Gulyás K. Polyák Á. Kovács K. J. (2017). Impaired microglia fractalkine signaling affects stress reaction and coping style in mice. Behav. Brain Res. 334, 119–128. 10.1016/j.bbr.2017.07.023 28736330

[B48] Xu B. Qian Y. Zhao Y. Fang Z. Tang K. Zhou N. (2019). Prognostic value of fractalkine/CX3CL1 concentration in patients with acute myocardial infarction treated with primary percutaneous coronary intervention. Cytokine 113, 365–370. 10.1016/j.cyto.2018.10.006 30352758

[B49] Yeom M. Ahn S. Oh J.-Y. Kim S.-Y. Lee H. Hahm D.-H. (2020). Atopic dermatitis induces anxiety- and depressive-like behaviors with concomitant neuronal adaptations in brain reward circuits in mice. Prog. Neuropsychopharmacol. Biol. Psychiatry 98, 109818. 10.1016/j.pnpbp.2019.109818 31743694

