# Supplementary material for: Adolescent stress and alcohol are associated with CX3CR1-linked endocrine–cardiac signatures and anxiety-like behavior in mice
Source: Front Pharmacol. 2026 Jun 24;17:1850815. doi: 10.3389/fphar.2026.1850815 (PMC13341564; doi:10.3389/fphar.2026.1850815)
Supplement: Supplementary file 1 [file Table1.DOCX]

**Table S1.** Characteristics of TaqMan Assays selected for gene expression quantification (Thermo Fisher Scientific).

| **Gene symbol** | **Gene name** | **Assay ID** | **Transcripts** | **Amplicon length** |
| --- | --- | --- | --- | --- |
| *Cx3cl1* | chemokine (C-X3-C motif) ligand 1 | Mm00436454_m1 | 13 | 72 |
| *Cx3cr1* | chemokine (C-X3-C motif) receptor 1 | Mm02620111_s1 | 8 | 107 |
| *Ccl2* | chemokine (C-C motif) ligand 2 | Mm00441242_m1 | 16 | 74 |
| *Ccl5* | chemokine (C-C motif) ligand 5 | Mm01302427_m1 | 13 | 103 |
| *Ccl11* | chemokine (C-C motif) ligand 11 | Mm00441238_m1 | 12 | 78 |
| *Cxcl12* | chemokine (C-X-C motif) ligand 12 | Mm00445553_m1 | 19 | 85 |
| *Ccr2* | chemokine (C-C motif) receptor 2 | Mm99999051_gH | 8 | 60 |
| *Cxcr4* | chemokine (C-X-C motif) receptor 4 | Mm01996749_s1 | 5 | 144 |
| *Ackr3* | atypical chemokine receptor 3 | Mm04931206_s1 | 5 | 63 |
| *Tnfrsf1a* | tumor necrosis factor receptor superfamily, member 1a | Mm00441883_g1 | 11 | 82 |
| *Tnfrsf1b* | tumor necrosis factor receptor superfamily, member 1b | Mm00441889_m1 | 10 | 64 |
| *Il1r1* | interleukin 1 receptor, type I | Mm00434237_m1 | 6 | 63 |
| *Nfkb1* | nuclear factor of kappa light polypeptide gene enhancer in B cells | Mm00476361_m1 | 11 | 70 |
| *Nfkbia* | nuclear factor of kappa light polypeptide gene enhancer in B cells inhibitor, alpha | Mm00477798_m1 | 7 | 70 |
| *Agtr1a* | angiotensin II receptor, type 1a | Mm01957722_s1 | 2 | 138 |
| *Ace2* | angiotensin I converting enzyme (peptidyl-dipeptidase A) 2 | Mm01159006_m1 | 7 | 97 |
| *Mas1* | MAS1 oncogene | Mm00434823_s1 | 7 | 96 |
| *Nr3c1* | nuclear receptor subfamily 3, group C, member 1 | Mm00433832_m1 | 13 | 68 |
| *Nr3c2* | nuclear receptor subfamily 3, group C, member 2 | Mm01241596_m1 | 3 | 85 |
| *Actb* | actin, beta | Mm02619580_g1 | 68 | 143 |
