# Supplementary figures and images for: Adolescent stress and alcohol are associated with CX3CR1-linked endocrine–cardiac signatures and anxiety-like behavior in mice
Source: Front Pharmacol. 2026 Jun 24;17:1850815. doi: 10.3389/fphar.2026.1850815 (PMC13341564; doi:10.3389/fphar.2026.1850815)

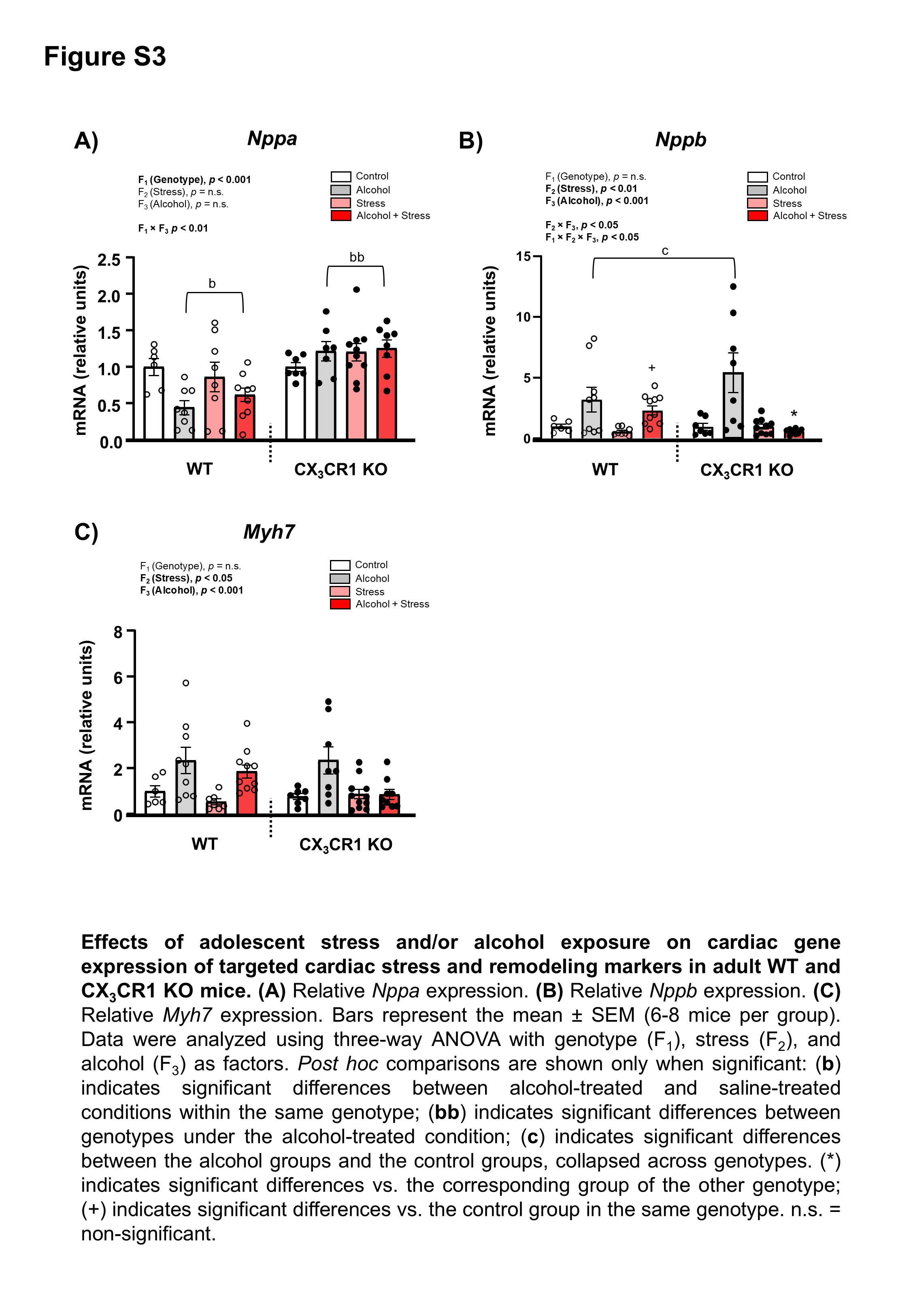

Supplement: Supplementary file 2 [file Image3.TIF]

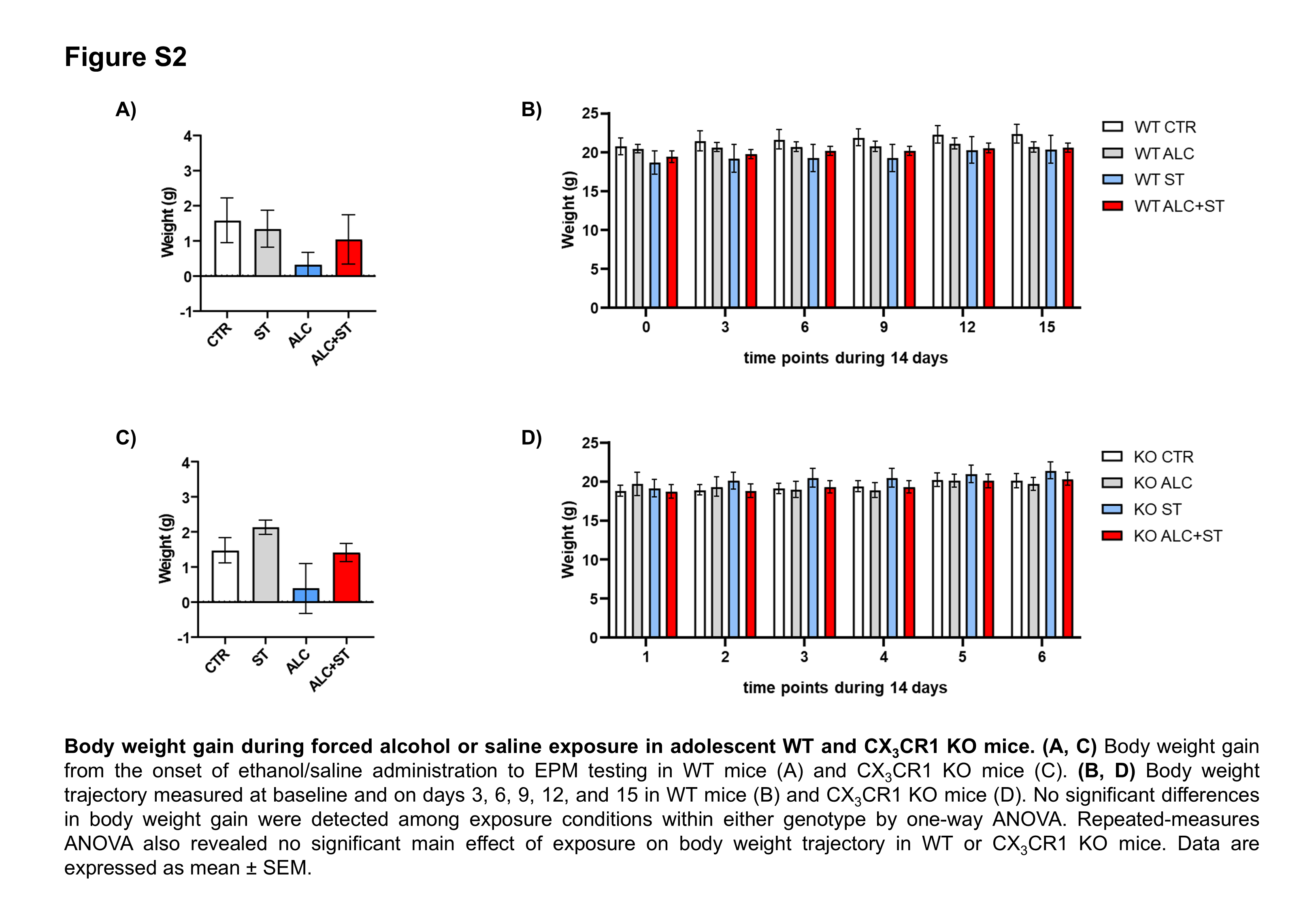

Supplement: Supplementary file 3 [file Image2.TIF]

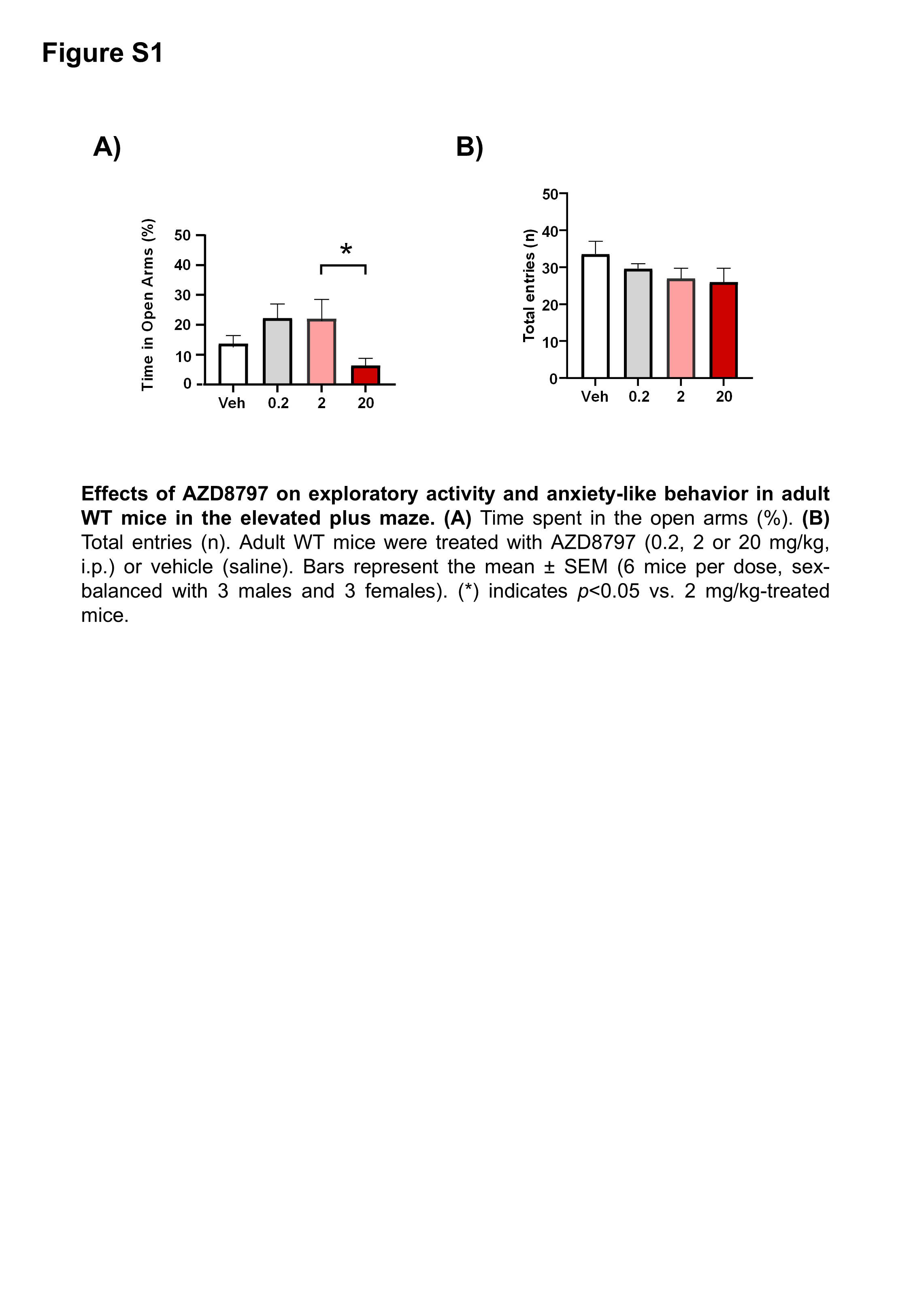

Supplement: Supplementary file 4 [file Image1.TIF]
