# Supplementary material for: Adolescent stress and alcohol are associated with CX3CR1-linked endocrine–cardiac signatures and anxiety-like behavior in mice
Source: Front Pharmacol. 2026 Jun 24;17:1850815. doi: 10.3389/fphar.2026.1850815 (PMC13341564; doi:10.3389/fphar.2026.1850815)
Supplement: Supplementary file 5 [file Table2.DOCX]

**Table S2.** Primer sequences used for the targeted qPCR analysis.

| **Gene symbol** | **Gene name** | **Forward primer 5’-3’** | **Reverse primer 5’-3’** |
| --- | --- | --- | --- |
| *Nppa* | natriuretic peptide A | TACAGTGCGGTGTCCAACACAG | TGCTTCCTCAGTCTGCTCACTC |
| *Nppb* | natriuretic peptide B | TCCTAGCCAGTCTCCAGAGCAA | GGTCCTTCAAGAGCTGTCTCTG |
| *Myh7* | myosin heavy chain 7 | GCTGGAAGATGAGTGCTCAGAG | TCCAAACCAGCCATCTCCTCTG |
| *Gapdh* | glyceraldehyde-3-phosphate dehydrogenase | CATCACTGCCACCCAGAAGACTG | ATGCCAGTGAGCTTCCCGTTCAG |
