# Supplementary material for: Adolescent stress and alcohol are associated with CX3CR1-linked endocrine–cardiac signatures and anxiety-like behavior in mice
Source: Front Pharmacol. 2026 Jun 24;17:1850815. doi: 10.3389/fphar.2026.1850815 (PMC13341564; doi:10.3389/fphar.2026.1850815)
Supplement: Supplementary file 6 [file Table3.DOCX]

**Table S3.** Descriptive plasma biomarker values collapsed according to the main factors included in the three-way ANOVA model for **Figure 3**.

| **Factor** | **Levels:** Experimental groups | **cTnI** | **cTnT** | **CX_3_CL1** | **ACTH** | **Corticosterone** |
| --- | --- | --- | --- | --- | --- | --- |
| **Genotype** | **WT** | 2.668 ± 0.063  n = 37 | 1.487 ± 0.057  n = 37 | 2.122 ± 0.015  n = 37 | 1.851 ± 0.040  n = 37 | 2.152 ± 0.036  n = 37 |
|  | **CX_3_CR1 KO** | 3.105 ± 0.073  n = 35 | 1.807 ± 0.067  n = 35 | 2.691 ± 0.014  n = 36 | 1.956 ± 0.031  n = 35 | 2.014 ± 0.046  n = 35 |
| **Stress** | **Non-stressed condition:**  Control and Alcohol groups | 2.875 ± 0.077  n = 32 | 1.569 ± 0.062  n = 32 | 2.358 ± 0.053  n = 33 | 1.956 ± 0.035  n = 32 | 2.189 ± 0.027  n = 32 |
|  | **Stress-exposed condition:**  Stress and Stress + alcohol groups | 2.886 ± 0.077  n = 40 | 1.702 ± 0.069  n = 40 | 2.439 ± 0.046  n = 40 | 1.858 ± 0.037  n = 40 | 2.002 ± 0.045  n = 40 |
| **Alcohol** | **Saline-treated condition:**  Control and Stress groups | 2.943 ± 0.079  n = 35 | 1.671 ± 0.070  n = 35 | 2.433 ± 0.049  n = 36 | 1.867 ± 0.040  n = 33 | 1.999 ± 0.044  n = 33 |
|  | **Alcohol-treated condition:**  Alcohol and Stress + alcohol groups | 2.822 ± 0.074  n = 37 | 1.616 ± 0.064  n = 37 | 2.373 ± 0.051  n = 37 | 1.931 ± 0.034  n = 39 | 2.158 ± 0.038  n = 39 |

Data are expressed as mean ± SEM of log10-transformed plasma concentrations. For genotype factor, values were collapsed across stress and alcohol exposure conditions. For stress factor, the non-stressed condition includes the control and alcohol groups, whereas the stress-exposed condition includes the stress and stress + alcohol groups. For alcohol factor, the saline-treated condition includes the control and stress groups, whereas the alcohol-treated condition includes the alcohol and stress + alcohol groups.
